# Informing conservation strategies with museum genomics: Long‐term effects of past anthropogenic persecution on the elusive European wildcat

**DOI:** 10.1002/ece3.8385

**Published:** 2021-12-03

**Authors:** Alina von Thaden, Berardino Cocchiararo, Sarah Ashley Mueller, Tobias Erik Reiners, Katharina Reinert, Iris Tuchscherer, Axel Janke, Carsten Nowak

**Affiliations:** ^1^ Conservation Genetics Group Senckenberg Research Institute and Natural History Museum Frankfurt Gelnhausen Germany; ^2^ Institute of Ecology, Evolution & Diversity Johann Wolfgang Goethe‐University, Biologicum Frankfurt am Main Germany; ^3^ LOEWE Centre for Translational Biodiversity Genomics (LOEWE‐TBG) Frankfurt am Main Germany; ^4^ Department of Physical Geography Johann Wolfgang Goethe‐University Frankfurt am Main Germany; ^5^ Senckenberg Biodiversity and Climate Research Centre Senckenberg Gesellschaft für Naturforschung Frankfurt am Main Germany

**Keywords:** archival DNA, bottleneck, conservation genetics, *Felis silvestris*, museum genomics, spatiotemporal data

## Abstract

Like many carnivore species, European wildcats (*Felis silvestris*) have suffered severe anthropogenic population declines in the past, resulting in a strong population bottleneck at the beginning of the 20th century. In Germany, the species has managed to survive its near extinction in small isolated areas and is currently recolonizing former habitats owing to legal protection and concerted conservation efforts. Here, we SNP‐genotyped and mtDNA‐sequenced 56 historical and 650 contemporary samples to assess the impact of massive persecution on genetic diversity, population structure, and hybridization dynamics of wildcats. Spatiotemporal analyses suggest that the presumed postglacial differentiation between two genetically distinct metapopulations in Germany is in fact the result of the anthropogenic bottleneck followed by re‐expansion from few secluded refugia. We found that, despite the bottleneck, populations experienced no severe genetic erosion, nor suffered from elevated inbreeding or showed signs of increased hybridization with domestic cats. Our findings have significant implications for current wildcat conservation strategies, as the data analyses show that the two presently recognized wildcat population clusters should be treated as a single conservation unit. Although current populations appear under no imminent threat from genetic factors, fostering connectivity through the implementation of forest corridors will facilitate the preservation of genetic diversity and promote long‐term viability. The present study documents how museum collections can be used as essential resource for assessing long‐term anthropogenic effects on natural populations, for example, regarding population structure and the delineation of appropriate conservation units, potentially informing todays' species conservation.

## Graphical abstract

European wildcats have suffered severe anthropogenic population declines in the past and are currently recovering their former habitats in Germany. Spatiotemporal genetic analyses based on historical and contemporary wildcat samples show that the presumed postglacial differentiation between metapopulations is in fact of recent anthropogenic origin. These findings have significant implications for current wildcat conservation strategies aiming to reconnect the populations by implementing migration corridors.

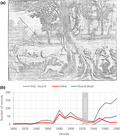

## 1 INTRODUCTION

The last decades have been characterized by an increasing and pervasive loss of biodiversity around the globe that has been mainly induced by human activities (Díaz, Settele, Brondízio, Ngo, Guèze, et al., 2019; Pimm et al., 2014). The associated anthropogenic impact on wildlife, however, and specifically the displacement of animals from their natural habitats has existed for a much longer time (Díaz, Settele, Brondízio, Ngo, Agard, et al., 2019).

While the extent of the resulting species’ loss or reduction is only now being fully acknowledged—for example, in many invertebrate communities—large mammals have been among the first to experience substantial population declines, geographic range contractions, and fragmentation of their habitats (Ceballos & Ehrlich, 2002; Morrison et al., 2007; Ripple et al., 2014). In contrast to large ungulates that were overexploited as game, large carnivores have been particularly affected by human–wildlife conflicts (Treves & Karanth, 2003), such as the extinct thylacine (Paddle, 2000). Perceived as competitors and imminent threats to human livelihoods, large carnivores have been extensively persecuted and consequently extirpated or driven to near extinction in most of Central Europe (Chapron et al., 2014). By the 1850s, several iconic species such as gray wolf, brown bear, and Eurasian lynx had been eradicated from major parts of the continent (Breitenmoser, 1998; Pereira & Navarro, 2015).

**FIGURE 1:**
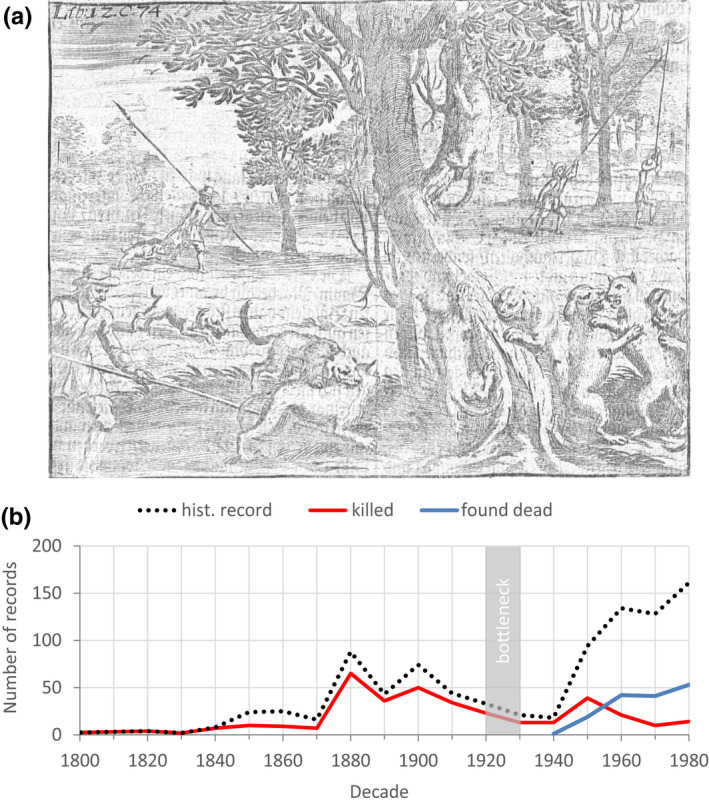
Anthropogenic persecution and historical records of European wildcats in Germany. (a) Hunt for disproportionally enlarged wildcats with sticks and dogs (undated copper engraving; von Hohberg, 1695). (b) Number of wildcats reported in historical records (dotted line), of which known cause of death was persecution (red line), or which were found dead (blue line, mostly road kills) in 1800–1980 (modified from Reinert, 2017). The gray rectangle represents the population bottleneck at its strongest magnitude according to Piechocki (1990)

The progressive disappearance of these apex predators was followed by population growth of herbivorous prey species, but also of medium‐sized carnivores and mesocarnivores (Prugh et al., 2009; Ripple et al., 2013, 2014; Ritchie & Johnson, 2009). At the same time, forest owners and hunters looking to replace profitable trophies with new prospects turned to hunt smaller carnivores such as the European wildcat (*Felis silvestris*, Schreber 1777) (Piechocki, 1990). The elusive carnivore was thence (mistakenly) held responsible for livestock damage and presented as a threat to humans, even if the animals’ body size, prey spectrum, and habitat needs did not fit this behavior (Figure 1a) (Müller‐Using, 1965). Following the proclamation of a trophy price for hunted wildcats in 1781, populations suffered from massive persecution and experienced intense range contraction (Figure 1b) (Reinert, 2017). Despite legal protection of the species through national legislations in the early 1900s, the European wildcat experienced a strong population bottleneck between 1920 and 1930 in Central Europe (Piechocki, 1990).

In Germany, wildcats were diminished to few remaining relict populations in low‐mountain refugia such as the Harz Mountains, the Palatinate Forest, or the Hesse Highlands (Piechocki, 1990). Eventually, the continued decline of wildcat populations was crucially—and positively—halted by the ban of steel snap traps in hunting in 1935 (Haltenorth, 1957).

In contrast to alarming global trends (Ripple et al., 2014), ranges of several large vertebrate species are expanding in Central Europe today (Cretois et al., 2020). The European wildcat serves as one successful example of rewilding densely populated European landscapes (Pereira & Navarro, 2015; Venter et al., 2016). Several factors have facilitated the recovery of wildcat populations, among others the reduced hunting pressure, increasing forest cover and progressive rural‐to‐urban migration of humans following World War II (Pereira & Navarro, 2015). Due to the species’ elusive nature and requirement for undisturbed broad‐leaved forest habitats, the re‐expansion of wildcats originating from secluded low‐mountain refugia was initially rather slow and may have even been overlooked in some areas for several decades (Piechocki, 1990).

The ongoing recolonization is currently monitored primarily using hair trapping and subsequent genetic detection (Steyer et al., 2013, 2016). Contemporary populations are estimated to comprise 5,000–8,000 individuals in Germany that occur predominantly along the low mountain ranges in the Central and Southwestern parts of the country and can be distinguished as two genetically divergent lineages or metapopulations (Mattucci et al., 2016; Steyer et al., 2016). The origin of this distinct spatial genetic pattern has occupied wildlife managers, stakeholders, and scientists for years as the two populations are located adjacent to each other, without separation by major barriers or an ecological gradient, and individuals from the current contact zone of the two populations hybridize (Mattucci et al., 2016; Steyer et al., 2016).

The recovery of wildcats following their near extermination represents a conservation success story, showcasing the effectiveness of legal protection in human‐dominated landscapes. However, the long‐term impact of the bottleneck on the species’ genetic composition remains unclear to date. Assessments of the preservation of local genetic variants and overall levels of genetic diversity as well as an evaluation of potentially increased hybridization with domestic cats have been missing.

To assess these possible anthropogenic effects on wildcat populations, we SNP‐genotyped and mtDNA‐sequenced historical and contemporary wildcat samples collected in Germany. Specifically, we investigate (i) if current (meta‐)population structure was caused by the bottleneck, (ii) whether populations experienced detectable genetic diversity loss, and (iii) if the extremely reduced population sizes have led to an increased prevalence of hybrids with domestic cats following the bottleneck.

Answering these questions is of fundamental importance for informing applied wildcat conservation strategies that are currently based on minimizing genetic diversity loss through the implementation of dispersal corridors (Mölich & Vogel, 2018) as well as monitoring regional levels of hybridization with domestic cats (Nussberger et al., 2018).

## 2 METHODS

### 2.1 Sampling

A combination of museum specimens (hereafter referred to as “historical”) and samples from extant German wildcat populations (“contemporary”) were used in this study (Figure S1). Historical samples of European wildcats from Germany (1830–2001; *n* = 175) were collected in 23 zoological museums in Europe (Table S1, sample details in Table SXL1, Appendix S2). Sample material consisted of preserved skins, footpads, fragments of skeletal bones or turbinal bones from the nasal cavity, teeth, and dried remains of tissue found on and in skulls. Contemporary samples originated from wildcat monitoring from 2006–2018 (*n* = 650) and represent the current wildcat distribution in Germany. These samples encompassed mainly tissues from road kills, as well as blood and noninvasively collected hair samples (Table SXL2, Appendix S2).

For most data analyses, samples were sorted into three temporal groups (prebottleneck, pre‐BN, 1830–1930; postbottleneck, post‐BN, 1931–2005; extant, 2006–2018) and two geographical groups (Western and Central metapopulation; hereafter referred to as “West” and “Central”). Temporal periods were delimited based on recorded year of sampling. The height of the bottleneck was assumed as 1930 (following Piechocki, 1990). The year 2005 was used as a limit to separate historical from contemporary samples, as the early 2000s represent the approximate time of resurging large‐scale research activities on wildcats in Germany. Consequently, genetic samples documenting the species’ expansion are increasingly available since this year. Samples were assigned a priori to geographical groups (metapopulations) following Steyer et al., 2016. For analyses in which Central and West were assessed separately, contemporary samples from the extant contact zone of the two populations (*n* = 26) were excluded in order to obtain reliable population‐level values. Further, 27 historical samples with ambiguous locality within Germany or imprecise sampling year were excluded from analyses after thorough verification of available collection information (labeled “NA” in Table SXL1, Appendix S2).

### 2.2 Sample preparation and DNA extraction

Special precautions were taken for obtaining DNA from historical samples. All laboratory procedures preceding PCR amplifications were conducted in a physically isolated low‐DNA environment, using a laminar flow hood solely designated to the handling of museum samples and including UV‐light filtering. Working equipment was cleaned with bleach between each sample preparation and radiated with UV for ≥40 min. Extraction batches included a maximum of eleven samples of the same material type and negative controls to monitor possible contamination. Skin, dried tissue remains, and footpads were cut into pieces of 20–70 mg and washed twice with 96% ethanol at 900 rpm for 15 min to remove chemicals from the preservation processes, followed by 30 min of drying at 50°C. Subsequently, samples were washed twice in double‐distilled H_2_O (PCR‐grade). Bones (20–170 mg), turbinal bones (20–80 mg), and teeth (7–150 mg) were additionally washed with an aqueous solution containing bleach for 10 min at 900 rpm before the washing steps described above. After washing, turbinal bones were crushed in reinforced 2 ml tubes with one ceramic bead of 6.8 mm diameter (Peqlab, Precellys^®^Keramik‐Kit CK68R) using a TissueLyser mill (Qiagen) in two consecutive steps of 30 sec at 30 Hz. Less fragile bone fragments and teeth were crushed for three consecutive runs of 10 s at 25,000 rpm, followed by 30 s at 25,000 rpm using an IKA^®^ Tube mill 100 with disposable grinding chambers including a stainless steel beater (MT‐40 sterile, IKA). The consecutive milling steps were alternated with small breaks of 30 s to prevent overheating of the biological materials. After sample preparation, all historical sample materials were extracted using the QIAamp DNA Investigator Kit (Qiagen) following the manufacturers’ protocol for buccal swabs with slight modifications: Lysis was performed with 400 µl ATL buffer, 25 µl proteinase K and 25 µl DTT. After lysis, 1 µl of carrier RNA was added to each lysate. DNA was eluted in 80 µl ATE buffer (Qiagen). Contemporary tissue and blood samples were extracted using the DNeasy Blood & Tissue Kit (Qiagen) and hair samples using the QIAamp DNA Investigator Kit (Qiagen). No animals were harmed or killed for this study, and all samples were collected in compliance with the respective local and national laws.

### 2.3 Molecular methods

#### 2.3.1 SNP genotyping

All historical and contemporary samples were genotyped for 96 SNPs selected for genetic assessments of European wildcat populations (von Thaden et al., 2020). The SNP panel contained two Y‐linked markers for sex identification (SRY SNPs), 10 SNPs selected for maximized *F*_ST_ between domestic and wildcats for detection of recent hybridization (HYB SNPs) and 84 markers with high heterozygosity values for individual identification (ID SNPs; von Thaden et al., 2020). SNP genotyping was performed on 96.96 Dynamic Arrays (Fluidigm) as described in von Thaden et al., 2020. Historical and contemporary samples were run in separate PCRs. While all experiments included a minimum of four no template controls to monitor for potential contamination, historical samples were additionally run with two positive controls (historical lynx samples). Before genotyping, samples were pre‐amplified (so‐called specific target amplification, STA) according to sample type: Historical samples were run for 28 cycles using 4 µl of DNA extract, while hair samples were run for 28 cycles with 3.2 µl of DNA extract and invasive samples (blood, tissue) were run for 14 cycles using 2 µl of DNA extract. Historical samples were at least triplicated, while only 10% of the contemporary tissue and blood samples were triplicated for estimation of potential genotyping errors. Contemporary hair samples were duplicated and only supplemented with a third replicate if the first two replicates showed a disagreement in their genotypes. Consensus genotypes of replicated samples and SNP genotyping error rates were determined as described in von Thaden et al., 2020. All samples with missing data rates of >15% were excluded from further analyses (*n* = 99 historical samples).

#### 2.3.2 Mitochondrial DNA

For mitochondrial DNA (mtDNA) sequence analysis, extracts of all historical samples were amplified with primers LF4 (5′‐GACATAATAGTGCTTAATCGTGC‐3′, Eckert et al., 2010) and H16498 (5′‐CCTGAAGTAAGAACCAGATG‐3′, Kocher et al., 1989) for a highly variable ~130 bp long fragment of the control region. PCRs were carried out in a total volume of 10 µl with 5 µl of 2X Multiplex PCR Master Mix (Qiagen), 0.5 µl of each 10 µM primer, and 3 µl of DNA extract. Following 15 min of denaturation at 95°C, 36 cycles with 30 s at 94°C, 90 s at 54°C, and 90 s at 72°C were run. Final extension was conducted for 10 min at 72°C. PCR products were purified and sequenced as described in von Thaden et al., 2017. All PCR setups included a minimum of four negative and two positive controls. Two samples of a historical lynx specimen were used as positive control, as these would amplify with the primers, resulting sequences were easy to distinguish from the target species’ sequences, and the samples featured similar quantities and qualities of DNA. All historical samples were run as duplicates in PCRs and sequenced at least twice in forward and reverse direction before building a consensus sequence for a sample. MtDNA data for extant populations were taken from Steyer et al. (2016).

### 2.4 Genetic data analyses

#### 2.4.1 SNP analyses

All genotyped individuals were screened for possible hybrids and domestic cats based on the 10 diagnostic HYB SNPs contained in the marker panel (von Thaden et al., 2020) using the Bayesian clustering methods implemented in NewHybrids v1.1 beta (Anderson & Thompson, 2002) and STRUCTURE v2.3.4 (Pritchard et al., 2000). NewHybrids was run under the uniform prior for 200,000 MCMCs, after discarding an initial burn‐in of 100,000 sweeps. In STRUCTURE, 200,000 MCMCs were preceded by a burn‐in of 30,000 steps, assuming correlated allele frequencies under the admixture model. Ten iterations of *K* = 2 were combined using CLUMPP (Jakobsson & Rosenberg, 2007). To aid clustering, 22 genotypes of reference domestic cats from von Thaden et al., 2020, were included in these analyses. Subsequently, all domestic cats, potential hybrids, and individuals with an assignment value for wildcats *q* ^(wc)^ ≤ 0.85 (historical, *n* = 15; contemporary, *n* = 42) were excluded from further analyses (genetic diversity and structure), leaving 56 historical and 608 contemporary wildcat individuals.

Population structuring was assessed based on the genotyped 84 ID SNPs using three clustering methods and analyzing historical and contemporary samples together. STRUCTURE was run for 10 iterations of 200,000 MCMCs after an initial burn‐in of 100,000 for *K* = 1–15 under the same assumptions as described before. The Evanno method (Evanno et al., 2005) implemented in STRUCTURE harvester (Earl & vonHoldt, 2012) was used to select the most likely *K*‐value. Replicate runs were combined using the LargeKGreedy algorithm of CLUMPP (Jakobsson & Rosenberg, 2007). Principal coordinates analysis (PCoA) was conducted as implemented in GenAlEx v6.502 (Peakall & Smouse, 2012). Additionally, a discriminant analysis of principal components (DAPC) was performed using the R package Adegenet v2.1.1 (Jombart, 2008). Groups for DAPC were assigned according to temporal and geographical classifications.

Genetic differentiation between the populations and sample groups was estimated based on pairwise *F*_ST_ values calculated with 5,000 permutations (Weir & Cockerham, 1984) in Arlequin v3.5 (Excoffier & Lischer, 2010). To calculate pairwise *F*_ST_ values between inferred population clusters (*K*) from STRUCTURE, samples with *q* ^(i)^ < 0.8 to any cluster were excluded. Genetic variability and diversity parameters (observed and unbiased expected heterozygosities, inbreeding coefficient, global pairwise *F*_ST_) and analysis of molecular variance (AMOVA) were calculated using GenAlEx v6.502 (Peakall & Smouse, 2012) and Arlequin v3.5 (Excoffier & Lischer, 2010). Differences in unbiased expected heterozygosity (u*H*_E_) between temporal and geographical groups were tested for statistical significance using the *wilcox*.*exact()* function in R (R Development Core Team, 2013) after ascertaining non‐normal distribution using the *shapiro*.*test()* function.

The Genhet v3.1 function (Coulon, 2010) was used to test for changes in SNP‐based genetic diversity over time and to calculate individuals’ standardized observed heterozygosity. Linear regressions in R were used to assess this parameter's relationship with time. Correlation between genetic and temporal distances was tested for West and Central following Casas‐Marce et al. (2017) and used to illustrate the intensity of allelic frequency changes over time. To do this, a matrix for linearized inter‐individual pairwise distance *â* (Rousset, 2000) was calculated using SPAGeDi v1.5 (Hardy & Vekemans, 2002) and correlated to a temporal distance matrix in years. A Mantel test with 9,999 permutations was used to assess statistical significance of the regression slopes, respectively.

#### 2.4.2 Mitochondrial sequence analyses

Sequence alignments were performed using Geneious v7.1.8 (Kearse et al., 2012) and haplotypes were assigned following Steyer et al. (2016). Historical samples with SNP genotypes assigned to domestic cat (see above) were excluded (*n* = 12) from all subsequent analyses based on mtDNA data (compare Methods in Appendix S1). The remaining samples were sorted into geographical (West, Central) and temporal groups (pre‐BN, post‐BN, extant) as described earlier. For comparison of haplotype prevalence and to account for the variable sample sizes, haplotype frequencies were calculated for the historical groups (this study) as well as for extant populations (data from Steyer et al., 2016). In order to simplify the depiction of haplotype frequencies over time, only haplotypes that were also found in the historical samples or show an extant frequency of >0.5% were taken from the modern dataset for comparison of frequencies (ten haplotypes in total; H03‐H07, H16, H22, H23, H40, H46). A temporal mtDNA haplotype network was constructed using the TempNet script (Prost & Anderson, 2011) in R (R Development Core Team, 2013).

## 3 RESULTS

### 3.1 Genotyping success and error rates

Complete mtDNA haplotypes (130 bp of the control region; min. four replicates) were obtained for 94 of 175 historical samples (53.7%). Acceptable SNP genotypes (min. three replicates, SNP call rate >85%) were achieved for 76 samples (43.4%), while 90 samples failed to amplify. Genotyping success varied between sample types, with calcified samples (bone and dental material) amplifying better on average (Table SXL3, Appendix S2), as has previously been reported (Dabney et al., 2013; Rohland & Hofreiter, 2007; Yang et al., 1998). Although mtDNA markers generally amplified better than SNP markers (10.3% higher amplification success), no mtDNA haplotypes could be obtained for ten samples that had been successfully SNP genotyped (13.2%). In contrast to the historical samples, all contemporary samples were genotyped successfully for both marker types without exception. The mean allelic dropout rate in SNP genotyping was 5.94% in historical and 0.22% in contemporary samples when comparing the replicates with consensus genotypes (Tables SXL4‐5, Appendix S2). False allele rates were 0.36% and 0.03% for historical and contemporary samples, respectively.

### 3.2 Genetic population structure and differentiation

**FIGURE 2:**
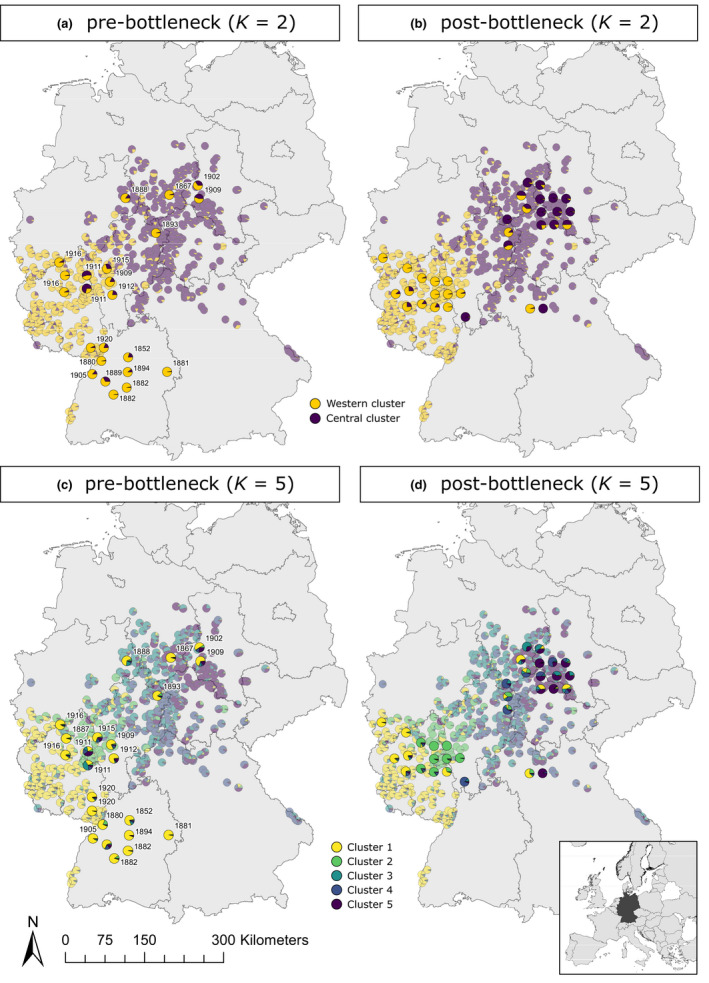
Spatial genetic population structure for historical and contemporary German wildcat samples. Results are shown separately for historical samples from pre‐ (a, c) and postbottleneck (b, d) periods, with results for extant wildcats (*n* = 608) shown in the background of each map as reference (transparent pie charts). Each individual sample is represented by a pie chart, in which colors indicate the likelihood of assignment (*q* ^(i)^) to the inferred genetic clusters. *K* = 2 (a, b) was identified as the most likely *K* as calculated with the Evanno method based on genotypes of 84 SNPs, followed by the second most likely *K* = 5 (c, d). Years in (a, c) correspond to the year of sample origin

Clustering analyses revealed changes in spatial genetic structure within the German wildcat populations through time (Figure 2). When analyzing historical and contemporary samples using STRUCTURE, the populations were subdivided into two clusters at *K* = 2 (Δ*K* = 200), largely fitting to the previously described Western and Central metapopulations.

However, historical samples collected before the 1930’s bottleneck (pre‐BN) did not show any spatial differentiation. Here, the analyzed individuals were mostly assigned to the contemporary Western cluster, even when collected from the Eastern part of the study area (Figure 2a). In contrast, postbottleneck (post‐BN) samples indicated signs of increasing differentiation into a discernable Central population (Figure 2b). This was also apparent when analyzing the second most likely *K* = 5 clusters (Δ*K* = 30) for all samples together (Figure 2c,d). At this level, fine‐scale structuring was revealed among the post‐BN and contemporary samples, separating the populations into wildcats from a far Western cluster (1, yellow), a Taunus cluster (2, light green), a Weser Uplands cluster (3, turquoise), a Hesse Highlands cluster (4, dark blue), and a Harz cluster (5, dark purple) (for geographical reference, see Figure S2). The extent of pairwise differentiation between the identified genetic STRUCTURE clusters was highest between samples from the Harz and the Taunus clusters (*F*_ST_ = 0.16) and lowest between samples from the Harz compared to the Hesse Highlands (*F*_ST_ = 0.06; Table S2, Appendix S1). Differentiation between the Western and Central metapopulation clusters was low (*F*_ST_ = 0.07 for *K* = 2).

When including samples from other wildcat populations in Europe (data from von Thaden et al., 2020) in the analyses, the increasing differentiation of the Central metapopulation continued to prevail (Figure S3). Both German pre‐BN sample sets and all samples from the Western metapopulation appeared more similar to contemporary Romanian, Italian, and Belgium wildcat individuals than to the post‐BN and extant Central population samples.

Grades of genetic differentiation between pairs of geographical and temporal groups were all significant (Table 1, *p  < *.05). Levels of differentiation were in accordance with our aforementioned findings of an increasing differentiation between Western and Central metapopulations following the bottleneck (pre‐BN, *F*_ST_ = 0.03–0.06; post‐BN, *F*_ST_ = 0.05–0.06; extant, *F*_ST_ = 0.06). Global *F*_ST_ values for temporal groups followed the same trend (Table 2).

**TABLE 1:** Pairwise genetic differentiation between temporal and geographical groups

		West			Central		
		Pre‐BN	Post‐BN	Extant	Pre‐BN	Post‐BN	Extant
West	Pre‐BN (*n* = 18)	–	.00	.00	.04	.00	.00
	Post‐BN (*n* = 14)	0.04	–	.03	.00	.00	.00
	Extant (*n* = 187)	0.03	0.01	–	.00	.00	.00
Central	Pre‐BN (*n* = 5)	0.03	0.06	0.04	–	.04	.00
	Post‐BN (*n* = 19)	0.06	0.06	0.05	0.03	–	.00
	Extant (*n* = 395)	0.06	0.06	0.06	0.06	0.02	–

Note

Pairwise *F*_ST_ values below diagonal, *p*‐values above diagonal. *F*_ST_ values were assumed as significantly different from zero based on *p* < .05; 5,000 permutations.

Abbreviations: Pre‐BN, prebottleneck; Post‐BN, postbottleneck.

**TABLE 2:** Historical and contemporary SNP diversity and differentiation in German wildcat populations

Epoch	Metapopulation	*n*	Dates range	*H*_O_	u*H*_E_	*F _IS_*	*p*‐value	*F*_ST_ global	*p*‐value
Pre‐BN		23	1852–1920	0.43 (±0.01)	0.46 (±0.01)	0.07	.07	0.02	.15
	West	18	1852–1920	0.42 (±0.01)	0.45 (±0.01)	0.08	.08		
	Central	5	1867–1909	0.47 (±0.02)	0.47 (±0.01)	−0.02	.54		
Post‐BN		33	1940–2001	0.41 (±0.01)	0.46 (±0.01)	0.09	.01	0.05	.00
	West	14	1940–2001	0.42 (±0.02)	0.43 (±0.01)	0.07	.16		
	Central	19	1951–1997	0.40 (±0.02)	0.44 (±0.01)	0.10	.04		
Extant		582	2006–2018	0.43 (±0.01)	0.46 (±0.01)	0.04	.00	0.06	.00
	West	187	2006–2018	0.44 (±0.01)	0.46 (±0.01)	0.05	.00		
	Central	395	2008–2018	0.43 (±0.01)	0.44 (±0.01)	0.03	.01		

Abbreviations: *F*_IS_, population inbreeding coefficient; *F*_ST_, genetic differentiation coefficient; *H*_O_, observed heterozygosity; *n*, number of individuals; u*H*_E_, unbiased expected heterozygosity; Pre‐BN, prebottleneck; Post‐BN, postbottleneck.

According to AMOVA, the amount of molecular variance between the metapopulations increased from 2% (pre‐BN) to 6% in post‐BN and extant populations (Table S3). Generally, most of the variance (86–95%) was found between individuals, owing to the SNP marker set being designed primarily for individual identification. Between temporal groups, molecular variance appeared highest (4%) when comparing pre‐BN to post‐BN samples and less (1%) when comparing post‐BN to extant samples both in the Western and Central metapopulations.

Several complementary analyses were conducted to verify the increasing differentiation of metapopulations after the bottleneck and to test for the influence of potential sampling effects on the analyses (see details in Methods and Results in Appendix S1). None of the results disagreed with the general finding of a post‐BN differentiation from the initial analyses (Figures S4‐S7, Tables S4‐S10). Further, results of other clustering methods corresponded with the STRUCTURE results, as pre‐BN Central samples clustered with Western population samples rather than extant Central samples (PCoA, Figure S8 and DAPC, Figure S9).

### 3.3 Genetic diversity through time

The comparison of SNP diversity resulted in highly similar heterozygosity values (*H*_O_, u*H*_E_) for all temporal and geographical groups (Table 2). The only significant differences (*p* < .001) were detected between pre‐BN Central populations (u*H*_E_ = 0.47 ± 0.01) and temporally subsequent central groups (post‐BN and extant, u*H*_E_ = 0.44 ± 0.01), as well as with the pre‐BN Western group (u*H*_E_ = 0.45 ± 0.01, Figure S10). Values for the population inbreeding coefficient *F*_IS_ (Table 2) were not significant for pre‐BN samples (*F*_IS_ = 0.07 for all groups), highest for post‐BN samples (especially in Central population, *F*_IS_ = 0.10, *p* < .05) and lowest for extant samples (*F*_IS_ = 0.03–0.05, all significant *p* < .05).

**FIGURE 3:**
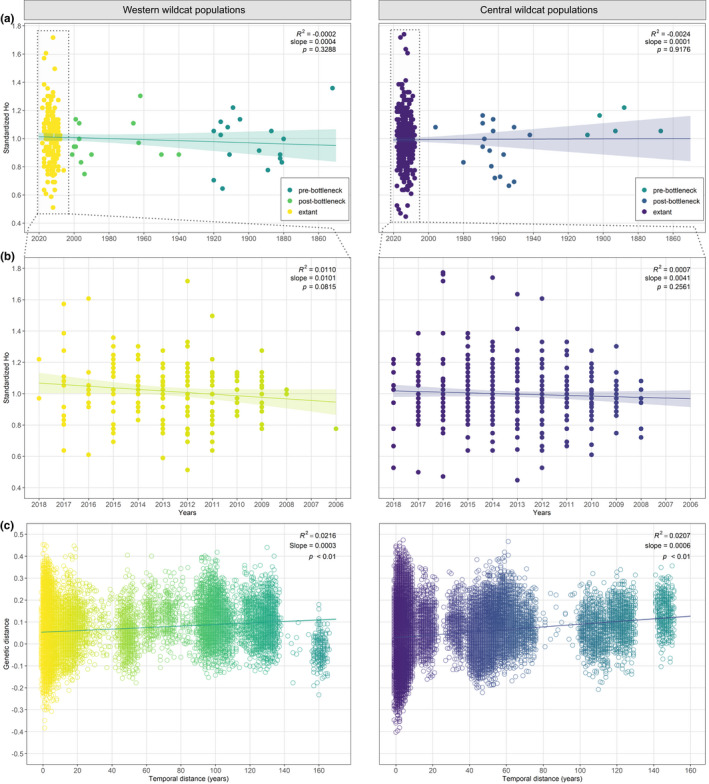
Genetic diversity and distance in German Western and Central wildcat populations over time. (a) Individual standardized observed heterozygosity (*H*_O_) for extant and historical wildcat populations over time, with (b) extant populations only (dotted lines indicate enlarged section from (a)). Heterozygosity over time was tested for a potentially significant decrease in diversity, as expected for populations that experienced strong bottleneck events. (c) Correlation of genetic distance (relatedness) versus temporal distance for pairs of individual samples (following Casas‐Marce et al., 2017). Genetic drift is represented by an increase in relatedness over time

Within the two metapopulations, the analyses of genetic diversity and distance over time did not evince an obvious effect of genetic drift (Figure 3). A slight increase of standardized *H*_O_ was discernable for the past ~150 years (not significant) (Figure 3a). This trend of increasing heterozygosity became more apparent when focusing on the extant samples in detail (Figure 3b). Here, the increase in genetic diversity appeared slightly higher in Western compared to Central populations (both not significant). However, when analyzing subsets of samples with equal sample sizes for each temporal period (Methods and Results in Appendix S1, Figure S11), Central wildcat populations seemed to have experienced a slight decrease in *H*_O_ (not significant).

Finally, the genetic similarity between pairs of individuals increased with time (Figure 3c) in a significant linear relationship (*p* < .01), indicating slight changes in allelic frequencies over time in both metapopulations (for subsets, see Figure S12).

### 3.4 Temporal change of haplotype frequencies

**FIGURE 4:**
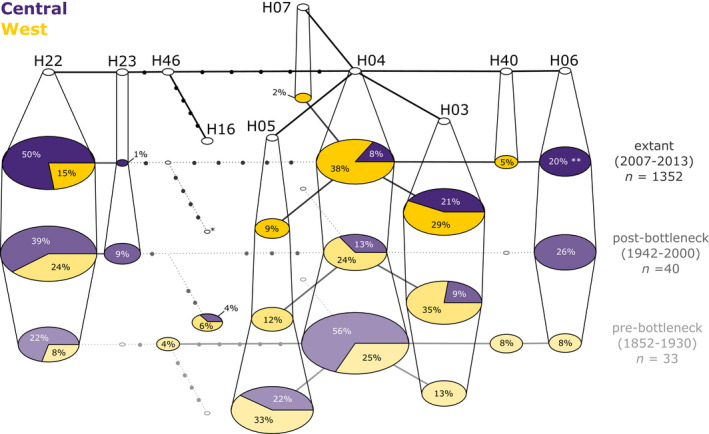
Temporal haplotype network for historical and extant German wildcats. Each circle represents one haplotype whose name is given in the uppermost layer. The following layers represent frequencies of these haplotypes at different temporal periods: extant (data from Steyer et al., 2016), post‐ and prebottleneck (historical museum samples from this study). The height of the bottleneck was assumed for the year 1930 (following Piechocki, 1990). Haplotype frequencies are indicated separately for Western (yellow) and Central (purple) geographical groups of German wildcat populations. The size of the circles reflects the percentage frequency of haplotypes. Small white circles indicate missing haplotypes for that time period. The number of dots plus the connecting haplotype equals to nucleotide differences. *, *n* = 1 sample in Western population (haplotype frequency of 0.15%); **, *n* = 3 samples in the Western population (haplotype frequency of 0.46%)

Before constructing haplotype networks, 21 mtDNA haplotype sequences were excluded due to ambiguous locality information or domestic cat classification (see Results in Appendix S1 and Table S1). All haplotypes found in the historical samples corresponded to common extant wildcat haplotypes, with no indication for a loss of maternal lineages (Figure 4). Differences in haplotype frequencies between post‐BN and extant populations appeared small (0%–15% per haplotype). However, when comparing historical haplotype frequencies from before the bottleneck (pre‐BN, Figure 4; bottom layer) to frequencies of extant populations (Figure 4; second highest layer), changes in geographical haplotype prevalence became apparent (0%–48% difference). Specifically, H05, which is exclusively found in the Western metapopulation in extant samples, appears in two samples from the pre‐BN Central populations (22% historical frequency). Also, haplotype H06, which is characteristic of the extant central metapopulation, is found twice in the pre‐BN western population (8% historical frequency), while appearing very rarely in extant western populations (0.46% extant frequency). Two samples from the post‐BN period (FS063, FS324), showed a common domestic cat haplotype (H16), but were classified as wildcats based on the 10 HYB SNP markers (Table SXL1, Appendix S2).

### 3.5 Hybridization assessment

**FIGURE 5:**
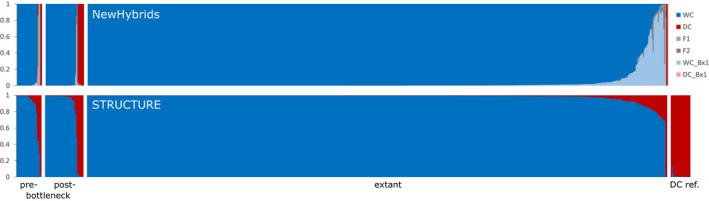
Identification of parental and hybrid individuals among historical and contemporary wildcat samples. Assessments were done using the software NewHybrids (top) and STRUCTURE (bottom) based on 10 SNPs selected for maximized *F*_ST_ between domestic and wildcats. For STRUCTURE, *n* = 22 reference domestic cats were included in the analyses to ensure proper clustering (DC ref.). WC, European wildcat; DC, domestic cat; F1, domestic × wildcat; F2, F1 × F1; WC_Bx1, F1 × wildcat; DC_Bx1, F1 × domestic cat

Several domestic cats and potentially admixed individuals were detected within both the historical and contemporary sample sets (Figure 5). Among the pre‐BN samples, two individuals were classified as domestic cats (FS326, FS344; both Central) and two individuals as potential F1 hybrids (FS044, West; FS320, Central). One individual (FS005, West) showed signs of admixture (*q* ^(wc)^ < 0.85), but could not be explicitly assigned to one of the genealogical classes. In the individuals sampled from the post‐BN period, seven were identified as domestic cats (FS088, FS093, FS094, FS095, FS119, FS121, FS122; all Central), one as potential F2 hybrid (FS029, West), and two individuals (FS019, FS020; both Central) could not be explicitly assigned to a genealogical class. Within the extant samples, two individuals were classified as domestic cats (FS0558f, FS3010f) and 40 samples showed varying degrees of admixture (*q* ^(wc)^ < 0.85). Results from STRUCTURE were congruent.

## 4 DISCUSSION

### 4.1 Genetic effects of the anthropogenic bottleneck

Strong population bottlenecks are one of the main drivers of genetic drift, often resulting in significant loss of genetic diversity. The extent of diversity loss and the associated consequences for population fitness are, however, not only dependent on the severity of the bottleneck but also its timescale (Hohenlohe et al., 2020). Both the duration and magnitude of the anthropogenic bottleneck in German wildcat populations are fairly well documented (Piechocki, 1990, and references therein), and resemble the demographic histories of other medium‐sized carnivores in Central Europe (e.g., Eurasian otter or European badger). Interestingly, even though population declines must have been severe until the height of the anthropogenic bottleneck in the early 20th century, we did not find evidence for a profound loss of genetic diversity, but rather a significant alteration in spatial genetic patterns. The most drastic changes were the temporal shifts in genetic population structure (Figure 2 and Figures S3‐S7) and the accompanying increase in spatial genetic differentiation between the two metapopulations over time (Table 1, Table S2). The results of the different clustering analyses suggest that pre‐BN individuals from the Central population resemble individuals of all time periods from the Western population more than their own postdecline progeny. Thus, the present metapopulations may have historically constituted a single, genetically diverse population with no major substructure.

The question whether the currently observed differentiation between a Western and Central metapopulation is based on the anthropogenically induced bottleneck or indeed much older and based on previously isolated glaciation relicts has occupied researchers and practitioners for years, as it has important implications for current conservation strategies (Eckert et al., 2010; Hertwig et al., 2009; Mattucci et al., 2016; Pierpaoli et al., 2003; Steyer et al., 2016). Several scenarios for the differentiation of the Central population have been discussed, namely (i) fast genetic drift after the strong anthropogenic bottleneck, (ii) rapid and ongoing range expansion that leads to edge effects and isolation‐by‐distance (IBD), (iii) refugial isolation during the Last Glacial Maximum (LGM), and (iv) different rates of introgression with domestic cats due to diverging population histories. In the following, we evaluate these hypotheses in light of genetic data obtained from historical populations in this study:

#### 4.1.1 Genetic drift

Genetic drift may stochastically lead to radical changes in allele frequencies and is particularly effective in association with bottleneck events (Amos & Harwood, 1998). In our wildcat example, genetic drift may have rapidly driven the differentiation between the Western and Central population, thus making several scenarios conceivable. For one, differences between the Western and the Central metapopulation could be explained by their geographical location. While both metapopulations must have suffered severely from the bottleneck, the Central population is situated at the Northeastern distribution edge of the species. Local refugia were rather scarce (e.g., Harz, northern Hesse Highlands) and geographically separated from the more Western areas of refuge (e.g., Palatinate Forest, Eifel, Hunsrück; Piechocki, 1990). Consequently, Central and Western populations must have been isolated for an extended period of time, leading to drift in small, bottlenecked local populations. However, in contrast to the isolated edge distribution of the Central population, the Western population is located adjacent to wildcat populations in France and Belgium. Connectivity to and gene flow from the adjacent populations may have mitigated the effects of postdecline genetic drift or even helped to maintain the historical level of genetic diversity (Jangjoo et al., 2016; Keller et al., 2001; McEachern et al., 2011). A recent study based on microsatellites found higher heterozygosity levels, as well as more private alleles and mtDNA haplotypes in the extant Western population, which supports this hypothesis (Steyer et al., 2016). In opposition, the Central population does not appear to be demographically connected to eastern European wildcat populations which could have counteracted drift effects (Hertwig et al., 2009).

Further, the Central population may have suffered stronger population size reductions due to its isolated geographical position or may have been exposed to long‐term, recurring fluctuations of their range and population size, which would have likely led to strong drift effects. However, we did not find clear evidence of significant decreases in heterozygosities in either metapopulation (Figure 3a,b, and Table 2), except for the five pre‐BN samples from the Central population (Figure S10). In the latter case, however, sample size is too low to make reliable assertions. Further, our findings may have been influenced by uneven sample sizes, as we did find a slight decrease of heterozygosity in the Central population (not significant) when analyzing subsets of samples with equal sample sizes (Methods and Results in Appendix S1, Figure S11). Genetic drift effects may have also acted more rapidly, continuing after the strong population decline due to human persecution. In this case, the effects of drift may be too subtle to identify in common genetic parameters, but pronounced enough to result in the observed spatial genetic patterns, similar to the extant subpopulation structures (Figure S5). Examples of such fast genetic drift exist for several recolonizing carnivores, such as the Central European wolf population (Szewczyk et al., 2019) or the reintroduced population of Eurasian lynx in the Harz Mountains (Mueller, Reiners, Middelhoff, et al., 2020).

**FIGURE 6:**
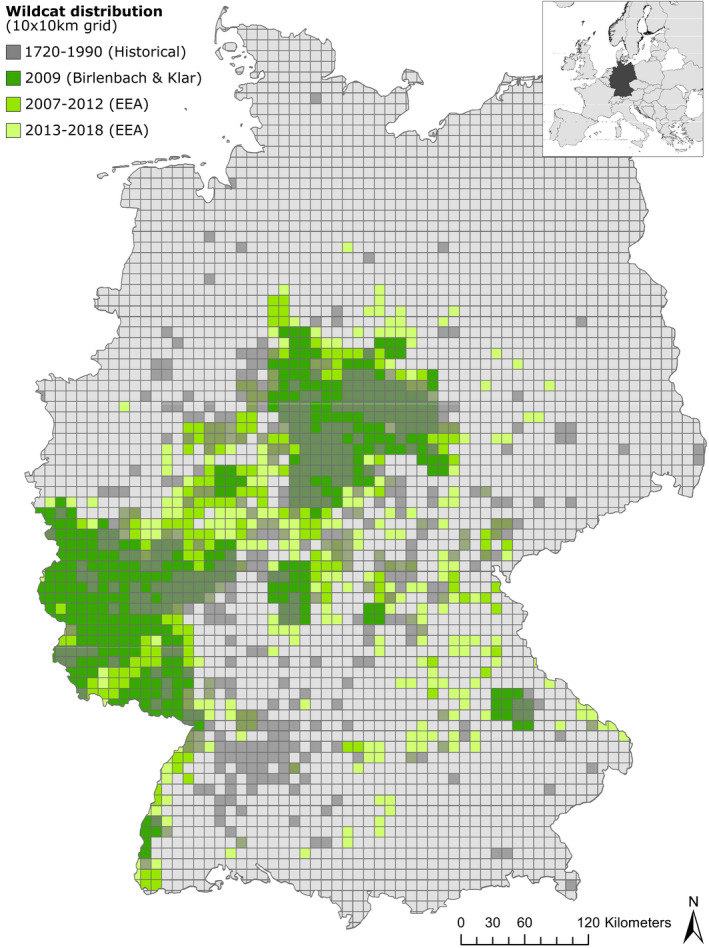
Historical distribution and ongoing range expansion of European wildcats in Germany. Comparison of historical records (dark gray) from Reinert (2017) to official contemporary distribution data (shades of green) from the European Environment Agency (EEA, 2020, Copenhagen, Denmark) and national reports (Birlenbach & Klar, 2009). Wildcat presence is depicted on a 10 × 10 km grid

The effects of drift were also noticeable in the mitochondrial sequence data, which suggest that haplotype frequencies have changed notably over time, whereas there was no sign of a complete loss of maternal lineages within the study region (Figure 4). This is surprising, as some of the historical populations that we analyzed seem to have been completely extirpated and their habitats not recolonized to date (e.g., the Black Forest in Baden‐Württemberg; cf. Figure 6). While this finding may suffer from sampling bias, it generally supports the hypothesis of a single, historically panmictic metapopulation.

#### 4.1.2 Edge effects in an expanding population

Another factor that could have shaped the metapopulation differentiation is distribution edge effects in the (re‐)expanding Central population (Williams et al., 2019). Range expansions usually involve a series of founder events which could have acted as a spatial analog of genetic drift in the Central population (Slatkin & Excoffier, 2012). Consequently, different forms of dispersal may generate distinct spatial patterns (Ibrahim et al., 1996), as could be the case here. Knowledge about the speed and dimension of wildcat range expansions following the bottleneck has been incomplete preceding the introduction of noninvasive genetic monitoring methods in the last few years (Steyer et al., 2013, 2016). Additionally, the presence of the species may have been overlooked for several decades, firstly because wildcats may be easily confused with domestic cats by untrained persons and secondly due to its elusive behavior (Steyer et al., 2013). Poorly documented reintroduction events further complicate the reconstruction, unless accompanied by traceable genetic traits (Mueller, Reiners, Steyer, et al., 2020). For example, the occurrence of haplotype H23 after the bottleneck (Figure 4) is clearly associated with reintroduction efforts in the Spessart low mountain range between 1984 and 2011 (Worel, 2009). Several of the >400 released individuals originated from Eastern Europe and thus explain the presence of this unusual haplotype in contemporary German wildcat populations (Steyer et al., 2016). Future fine‐scale studies will be needed to further elucidate the potential influence of edge effects, taking a standardized sampling and appropriate numbers of genetic loci into account.

#### 4.1.3 Glacial refugial isolation

Our findings are in contrast to earlier studies which hypothesized that the Central German metapopulation, which is genetically distinct from all other European populations, might have been the result of refugial isolation during the late‐Quaternary Last Glacial Maximum (LGM) (Mattucci et al., 2016; Steyer et al., 2016). Similar differentiation legacies have been found in numerous European species (e.g., brown bear, Davison et al., 2011; roe deer, Sommer et al., 2009; wild boar, Scandura et al., 2008; see also Hewitt, 1999; Schmitt, 2007). This view is questioned, however, by the absence of potential glacial refugia within the current species’ range, the lack of evidence for any morphological differentiation or the detection of private historical mitochondrial haplotypes (Sommer & Benecke, 2006). On the contrary, some of the haplotypes appear to be regionally private or distinctly predominant in the extant rather than the historical populations (e.g., West, H05; and Central, H06; Figure 4). The detection of these currently regional private haplotypes in other areas based on the pre‐BN samples demonstrates the considerable impact of the bottleneck on local maternal diversity. One possible explanation could be that the haplotypes were more prevalent once, that is, common in both metapopulations before the bottleneck, and that their occurrence was reduced to local populations through the decline, which is consistent with the nuclear genetic data (SNP results). However, bottlenecks may have very different influences on haplotypic diversity of naturally recovering populations (Sonsthagen et al., 2017), thus our findings can only be classified as indicative. Further, private haplotypes may have also been present in historical populations, but not been captured in the present study due to the limited number of historical samples.

Mattucci and colleagues conducted Approximate Bayesian Computing (ABC) simulations to assess the phylogeographic history of European wildcat populations and estimated a divergence time of ~30,000 years for the Central German metapopulation and other investigated Central European populations (i.e., Belgium, Luxembourg, Switzerland, and Western German metapopulation) (Mattucci et al., 2016). However, the uncertainty of their modal values (0.25–0.75 quantiles) was reportedly high and underlines the challenges associated with ABC inferences. While ABC methods offer a wider application of model‐based statistical inference than traditional Bayesian approaches, it has also been the subject of controversial debate in the scientific community (Berger et al., 2010; Robert et al., 2011; Templeton, 2009). Parameter estimation, model selection as well as corresponding assumptions and approximations heavily influence the outcome of the simulations and thus need to be carefully assessed and evaluated (Sunnåker et al., 2013). In order to take full advantage of the potentials of ABC methods in wildcat phylogeographic research, comprehensive future work should focus on the inclusion of data from ancient and historical specimens to further verify the models (Casas‐Marce et al., 2017), and may even take novel machine learning approaches like deep learning into account to optimize parameter selection (Mondal et al., 2019).

#### 4.1.4 Ancient or historical introgression

Introgressive hybridization is a natural phenomenon that may be either beneficial, neutral, or detrimental for the evolutionary trajectory of a species. Hybridization between wild‐living taxa and their domestic congeners, however, is usually judged as unfavorable (outside of targeted breeding), as the domestic taxa may genetically swamp their wild relatives and lead to genetic extinction of the latter (Tiesmeyer et al., 2020). Consequently, the assessment of hybridization represents a critical conservation issue. The current habitats of European wildcat populations are situated in landscapes that are densely populated by humans and their domestic cats (EPFI, 2019). Thus, the risks for hybridization between the two species are high and incidences have been reported throughout Europe (Beaumont et al., 2001; Lecis et al., 2006; Nussberger et al., 2014; O’Brien et al., 2009; Oliveira et al., 2008; Steyer et al., 2018; Tiesmeyer et al., 2020). In this light, different levels of ancient or historical introgression appropriated before or after the anthropogenic bottleneck could explain the differentiation of the currently observed metapopulation patterns in Germany. This hypothesis is supported by the fact that hybrids have been shown to occur more frequently at the periphery of wildcat ranges (Randi et al., 2001) and a high degree of habitat fragmentation may further enhance these edge effects (Tiesmeyer et al., 2020). In Switzerland, for instance, recent range expansions have led to increased local hybridization rates (Nussberger et al., 2018; Nussberger, Wandeler, Weber, et al., 2014). The theory of modern introgression is, however, contradicted by the fact that the contemporary German wildcat populations possess some of the lowest hybridization rates (Central, 3%; West, 5%; Steyer et al., 2018; Tiesmeyer et al., 2020). Accordingly, although we did find admixed individuals in all three temporal groups (Figure 5), there was no hint of an increased presence of hybrids in any of the groups. Even low, and potentially undetected, levels of introgression could, however, explain the slight increase in *H*_O_ in the contemporary samples (Figure 3b) and will need to be further elucidated. While our sample numbers in the historical groups are naturally not comparable to the investigated contemporary samples, it is also important to note that we assessed hybridization based on only 10 ancestry‐informative SNPs (von Thaden et al., 2020). More fine‐scale evaluations for introgression should incorporate higher SNP numbers, especially to distinguish different hybrid categories with high certainties (Mattucci et al., 2019; Nussberger et al., 2014; Oliveira et al., 2015; Steyer et al., 2018). As previous studies have suggested prehistoric gene flow between the ancestors of European wildcats and domestic cats, future studies focused on ancient introgression will probably be based on whole‐genome sequencing (WGS) data and might even take paleogenomic evidence into account in order to unravel these complicated phylogenetic relationships (Driscoll et al., 2007; Howard‐McCombe et al., 2021; Ottoni et al., 2017).

While we cannot rule out that any of the four abovementioned factors may have contributed to the origin of the observed spatial differentiation, we argue that it appears to be a direct consequence of the anthropogenically induced bottleneck. Based on our results, the existence of a single, genetically diverse and panmictic metapopulation preceding the anthropogenic persecution appears as the most likely scenario. The local wildcat populations have probably experienced a combination of genetic drift during refugial isolation and range edge effects during the re‐expansion following legal protection. Contemporary populations still carry the resulting spatial patterns in their genetic legacy, although Central subpopulations already seem to intermix in the course of their recolonization and consequently lose their distinct genetic substructuring (Figure S5). The differentiation between the Western and Central metapopulation, however, will probably take longer to fade out, mainly due to the geographically disjunct location of the current metapopulations.

### 4.2 Limitations and methodological issues

Surprisingly, we did not find any clear indication of genetic drift or other phenomena that accompany sharp reductions in population size. This may be explained by the type of bottleneck, as substantial genetic diversity loss may only become detectable if population sizes are below a certain threshold (e.g., *N* < 200 in Hoban et al., 2014). Hoban et al. (2014) conclude in their simulation study that the detection and monitoring of genetic erosion may be unfeasible ‐ even considering many genetic markers ‐ if the effective population size exceeds several hundreds. Although the latter seems unlikely for the wildcat, it cannot be excluded that the anthropogenic bottleneck event may not have been as severe as previously stated by Piechocki (1990) and/or that local wildcat populations might have been overlooked until recently. This is because standardized genetic monitoring, which represents the most effective detection method for this elusive species, only started in the early 2000s (Steyer et al., 2013). As a matter of fact, we did not find any signs of inbreeding (*F*_IS_ values; Table 2) or strong loss of genetic diversity.

Genetic data are commonly used to assess the timing of bottleneck events in conservation genetic studies, but the ability to reliably determine these is subject to several factors (Peery et al., 2012). While the timescale and severity of the bottleneck markedly influence the resulting genetic effects for a population or species (Hundertmark & Daele, 2010; Sonsthagen et al., 2017), they are also depending on the level of pre‐ and postdecline diversity, as ancient reductions in genetic diversity may mask recent declines (Dussex et al., 2015). More specifically, if genetic diversity is already low, for example, due to an ancient decline, before experiencing a recent bottleneck, genetic losses may be impossible to detect (Cornuet & Luikart, 1996; Dussex et al., 2015; Sonsthagen et al., 2017). Further, long‐lived species may be able to preserve genetic diversity over shorter periods of decline (Hailer et al., 2006; Johnson et al., 2008; White et al., 2014). Various methods exist for the detection of bottlenecks, ranging from classical tests for loss of heterozygosity and changes in allele distribution frequencies to more recent approaches based on genomic data (Allendorf, 2017; Cammen et al., 2018; Luikart et al., 1998). All of these methods rely on a series of assumptions and models, such as a model and rate for mutations, absence of gene flow, mutation‐drift equilibrium before decline, or uniformity of reproductive success (Gattepaille et al., 2013; Hoban, Mezzavilla, et al., 2013). Further, demographic reconstructions suffer from bias and confounding factors such as the presence of population structure (Peter et al., 2010; Sousa et al., 2012), insufficient sampling or the choice of marker systems including their associated ascertainment bias (Hoban et al., 2013; Williamson‐Natesan, 2006). As we could not exclude the violation of several of the above‐mentioned assumptions, we did not conduct bottleneck tests in this study but focused instead on the evaluation of spatial patterns. Based on our findings ‐ especially the lack of evidence for common genetic bottleneck effects ‐ subsequent studies are advised to incorporate a more equally distributed sampling scheme and probably a much higher number of markers (if not WGS data) to further elucidate the demographic history of European wildcats in Germany.

Reconstructing demographic histories based on genetic analyses of collection material involves both opportunities and challenges (Newbold, 2010). General limitations of data from museum resources may result from errors (e.g., ambiguous locality information or wrong species identity) and/or biases (e.g., spatial, environmental, temporal, and taxonomic) (Graham et al., 2004; Newbold, 2010; Soberón et al., 2000). Some of these limitations may be overcome and require careful scrutiny, which led us to exclude 27 of the collected historical specimens from the analyses. Further, regional and temporal biases often make representative, standardized sampling of a specific geographical or temporal group impossible, resulting in low sample numbers that hamper sound statistical inferences, for instance for the Central pre‐BN individuals (*n* = 5) in this study. One option to test for potential sampling effects lies in subsampling contemporary populations for more balanced sample sizes, as we did here (Methods and Results in Appendix S1, Figures S4‐S7 and S11‐S12, Tables S5‐S10). Concomitantly, time series do not exist for many species and populations, and are often heavily biased (see above). Considering the resulting gaps in our historical data, we may have missed parts of genetic diversity in the present assessments. Although heavily persecuted, killed wildcats have probably never reached the status of more prominent historical game species, such as the wolf, lynx, and red deer, and may have been considered less as a trophy worth of taxidermic preservation. This lesser interest in collecting wildcats for natural history museums may have influenced our findings and should be considered. For example, it seems possible that wildcats were already scarce before the mid‐19th century, that is, before the specimens in this study were collected, so that the bottleneck event may actually extent much farther into the past and consequently might have affected our results.

Another significant aspect is the choice of marker system and the number of loci used to detect genetic erosion (Hoban, Gaggiotti, et al., 2013; Peery et al., 2013). Here, we used a panel of 96 SNPs, of which 84 were selected for maximized heterozygosity in contemporary wildcat populations (von Thaden et al., 2020). The selection of highly polymorphic markers is typically associated with some degree of ascertainment bias, which may have affected the results in this study. Firstly, an optimized panel of polymorphic markers does not provide unbiased estimates of genetic indices, may potentially overestimate current diversity, and, owing to the restricted number of SNPs, does not represent genome‐wide diversity (Geibel et al., 2021). Secondly, the absence of signs of genetic erosion may stem from the SNP panel being designed using solely contemporary samples. Accordingly, the SNPs may not completely reflect historical diversity, potentially leading to underestimation of genetic losses, lower estimates of pairwise *F*_ST_ and lack of genetic structure in historical samples. Indication for masked historical diversity is, for instance, observable in the DAPC (Figure S9), where pre‐BN samples form an adjacent, but separate cluster as compared to extant individuals. Further, it is important to note that the typed SNPs are bi‐allelic (Albrechtsen et al., 2010; Malomane et al., 2018). Other than multi‐allelic microsatellites, bi‐allelic SNP markers are not as prone to exhibit loss of alleles. Consequently, the detected diversity changes in this study were limited to changes in allele frequencies rather than number of alleles, whereas the latter have been assessed as the best indicator for monitoring genetic erosion following declines with high statistical power (Hoban et al., 2014). We chose to use SNP markers because they have proven to yield higher amplification successes and lower error rates when genotyping degraded sample materials, also due to their short amplicon lengths (<120 bp; von Thaden et al., 2017, 2020). Indeed, the rates of allelic dropout and false alleles in this study were in the range of similar research work based on microsatellite genotyping (e.g., Casas‐Marce et al., 2017; Jansson et al., 2014). Nevertheless, to increase statistical power for the monitoring of genetic erosion in subsequent studies and reduce potential effects of ascertainment bias, we recommend to aim for a higher number of SNP markers (min. several hundred; e.g., Cammen et al., 2018; Ewart et al., 2019; Stronen et al., 2019a), consider second‐generation sequencing of microsatellites (Curto et al., 2019), or data from WGS (Larsson et al., 2019; Loog et al., 2020; White et al., 2018).

### 4.3 Conclusions and implications for conservation

European wildcats in Germany have survived centuries of population decline and massive anthropogenic persecution. Today, populations are expanding their ranges, appear to recolonize most of their former habitats, and even seem to advance to newly occupied areas (Figure 6, with details in Appendix S1 Methods and Results; Steyer et al., 2016; Reinert, 2017; Balzer et al., 2018). Based on the presence data alone, the extant wildcat populations appear viable and thriving and offer reason to hope for a successful re‐establishment of the species within the next decades into the presently remaining gaps of its historical distribution (Figure 6; Balzer et al., 2018). The long‐term viability of a species, however, is not only dependent on the sheer number of individuals, but also of the genetic makeup of its populations (Hoban et al., 2020). While the species’ recovery in a cultivated landscape can certainly be evaluated as a conservation success, genetic monitoring of wildcats is still required to assess postdecline development of wildcat populations (Steyer et al., 2016; Tiesmeyer et al., 2018). Our findings suggest that the anthropogenic bottleneck has significantly shaped the currently observed spatial genetic structuring. The bottleneck and subsequent founder effects during early re‐expansion likely led to the emergence of a genetically differentiated metapopulation, which appears to have been absent before the strong population size reductions. Although our analyses of historical and contemporary specimens have not revealed clear indication of a loss of diversity, inbreeding or increased hybridization, the extant populations appear to be genetically different to their historical ancestors.

Today, the European wildcat serves as an important flagship species in conservation strategies aiming to reconnect fragmented forest landscapes in Germany and other parts of Europe. Large‐scale long‐term conservation projects led by the Friends of the Earth Germany (BUND) aim at implementing forest corridors in different areas throughout the country to allow for effective connection of the disjunct wildcat populations (Mölich & Vogel, 2018). The main goal is to generate viable metapopulations and to support the creation of a biotope network that associated forest species will equally benefit from.

The present study corroborates these strategies and, for the first time, offers historical data to confirm the adequacy of these management plans from a genetic point of view. Our spatiotemporal analyses suggest that the differentiation between West and Central was the result of recent anthropogenic persecution followed by re‐expansion of the species. In consequence, the current metapopulations should genetically be treated as a single management unit. Further, our results indicate that the populations in Germany have neither suffered major losses of genetic diversity, nor experienced massive genetic erosion, inbreeding or increase in hybridization following the bottleneck. Although some of these findings can only be judged as indicative given the unequal sampling of historical specimens, they are generally in line with earlier findings (Balzer et al., 2018; Steyer et al., 2016, 2018) and will likely be revisited by future studies based on WGS. For now, the endangered wildcat populations in Germany appear under no imminent threat from genetic factors and consequently viable in long term. While an active reconnection of the disjunct populations is not absolutely essential from a genetic perspective, it will certainly facilitate the ongoing range expansion of the species. The resulting convergence of West and Central populations may promote the restoration of genetic diversity in German wildcat populations to levels seen before the onset of massive persecution.

In conclusion, our study demonstrates how the inclusion of historical genetic data, for example, from museum records, serves as an important tool to understand a species’ demographic history and take appropriate and effective conservation actions (Barnosky et al., 2017; Fenderson et al., 2020; Meineke et al., 2018).

## CONFLICT OF INTEREST

None declared.

## AUTHOR CONTRIBUTIONS

**Alina von Thaden:** Conceptualization (equal); data curation (lead); formal analysis (lead); investigation (equal); methodology (lead); validation (equal); visualization (lead); writing–original draft (lead); writing–review and editing (lead). **Berardino Cocchiararo:** Methodology (supporting); writing–review and editing (supporting). **Sarah Ashley Mueller:** Methodology (supporting); writing–review and editing (supporting). **Tobias Erik Reiners:** Methodology (supporting); software (supporting); writing–review and editing (supporting). **Katharina Reinert:** Data curation (supporting); formal analysis (supporting); methodology (supporting); software (supporting); writing–review and editing (supporting). **Iris Tuchscherer:** Data curation (supporting); formal analysis (supporting); methodology (supporting); writing–review and editing (supporting). **Axel Janke:** Conceptualization (equal); supervision (equal); writing–review and editing (supporting). **Carsten Nowak:** Conceptualization (equal); funding acquisition (lead); investigation (supporting); project administration (equal); supervision (equal); writing–original draft (supporting); writing–review and editing (supporting).

## Supporting information

Appendix S1

Appendix S2

## Data Availability

SNP genotyping data are available in the Dryad repository: https://doi.org/10.5061/dryad.31zcrjdmr. Sample information for this study is available in the Supplementary excel files (Tables SXL1‐2 in Appendix S2).
