# Supplementary material for: Informing conservation strategies with museum genomics: Long‐term effects of past anthropogenic persecution on the elusive European wildcat
Source: Ecol Evol. 2021 Dec 3;11(24):17932–51. doi: 10.1002/ece3.8385 (PMC8717334; doi:10.1002/ece3.8385)
Supplement: Supplementary file 1 — Appendix S1 [file ECE3-11-17932-s002.pdf]

Supplementary Information

Informing conservation strategies with museum genomics: long-term effects of past anthropogenic persecution on the elusive European wildcat

Alina von Thaden [a,b]*, Berardino Cocchiararo [a,c], Sarah Ashley Mueller [a,b], Tobias Erik Reiners [a], Katharina Reinert [a,d], Iris Tuchscherer [a,b], Axel Janke [b,c,e], Carsten Nowak [a,c]

[a] Conservation Genetics Group, Senckenberg Research Institute and Natural History Museum Frankfurt, Clamecystraße 12, 63571 Gelnhausen, Germany

[b] Institute of Ecology, Evolution & Diversity, Johann Wolfgang Goethe-University, Biologicum, Max-von-Laue-Straße 13, 60438 Frankfurt am Main, Germany

[c] LOEWE Centre for Translational Biodiversity Genomics (LOEWE-TBG), Senckenberganlage 25, 60325 Frankfurt am Main, Germany

[d] Department of Physical Geography, Johann Wolfgang Goethe-University, Altenhöferallee 1, 60438 Frankfurt am Main, Germany

[e] Senckenberg Biodiversity and Climate Research Centre, Senckenberg Gesellschaft für Naturforschung, Senckenberganlage 25, 60325 Frankfurt am Main, Germany

* Corresponding author:

Alina von Thaden, alina.vonthaden@senckenberg.de

Supplementary Methods and Results

Complementary SNP analyses

In order to verify the results of a post-bottleneck (post-BN) differentiation of the Western and Central metapopulations and to test for sampling effects, several complementary analyses were conducted, which are described in the following.

Genetic population structure and differentiation

Firstly, the potential influence of contemporary samples on the clustering of the historical samples was tested. To do this, the historical samples were analyzed separately for pre- (n = 23) and post-bottleneck (n = 33) periods using STRUCTURE, and excluding contemporary individuals. The clustering results confirmed a lack of considerable structure preceding the bottleneck event (Figure S4). Pre-bottleneck (pre-BN) samples showed only very low differentiation (ΔK = 7 for most likely K = 7) of four individuals from the Taunus region, while post-BN samples indicated an increase in sub-structuring (ΔK = 51 for most likely K = 2).

Secondly, the temporal sample sets were analyzed separated by their geographical origin, i.e. their geographical assignment to the Central and Western metapopulation, in order to test if the predominant differentiation between the two contemporary metapopulations had superimposed local fine-scale structuring in the historical populations (Figure S5). The results demonstrate that structuring increased in the Central population over time toward a contemporary differentiation of K = 3 (Figure S5, a, b). In the pre-BN Western samples, however, population structure appeared as initially absent, while a private cluster of individuals from the Taunus region could be delineated following the bottleneck (Figure S5, c, d). The contemporary Western samples show further differentiation into K = 3, with an additional distinction of wildcats from the Southern part of Palatinate. The highest pairwise differentiation was found between samples from the Southern parts of Palatinate to samples from the Taunus (F_{ST} = 0.2; Table S2),

whereas the Central metapopulation did not show high differentiation within its three population clusters (F_{ST} = 0.08-0.10).

Thirdly, to ascertain potential effects of sampling bias through unequal sample sizes in the SNP analyses (Wahlund, 1928), two types of subsets were created for which sample sizes were adjusted to 23 individuals in each temporal period (i.e., n =23 for pre-BN, post-BN, extant, respectively; Table S4). Subsets were selected following two approaches: (i) based on geographical proximity of the individuals collected in different periods in order to minimize influence from range distances, and (ii) randomly, using the *sample()* function in R (R Development Core Team, 2013). STRUCTURE results for the sample subsets (selected for 'geographic proximity' vs. 'random'; Figures S6-S7) corroborated the findings based on the full dataset (Figure 2): pre-BN samples of the Western and Central population clustered mostly with the extant Western samples at K = 2, while post-BN samples revealed signs of population differentiation. Results for K = 3 showed further differentiation of individuals from the Taunus region (light green cluster), which was also apparent with the full dataset (Figure 2, d). Pairwise genetic differentiation (F_{ST}; Tables S5-S6) and global F_{ST} values (Tables S7-S8) were in line with the trend of an increasing differentiation between Western and Central metapopulations following the bottleneck, although several values were not significant due to the lower sample numbers in the subsets. AMOVA analyses for the subsets also followed the trends seen with the full dataset (Tables S9-S10). In general, differences between the results from the two subsampling approaches ('geographic proximity' vs. 'random') appeared small.

Genetic diversity through time

SNP diversity showed similar heterozygosity values (H_O, uH_E) for temporal and geographical groups based on the sample subsets ('geographic proximity' vs. 'random') as compared to the results from the full dataset, while F_{IS} values were not significant (Tables S7-S8, full dataset in Table 2).

When analyzing individual standardized H_O over time for the two subsets, the results for the Western populations were congruent, showing very slight trends of increasing heterozygosity over time

(Figure S11). However, analyses for the Central population revealed a slight trend of decreasing heterozygosity over time in both subsets (not significant, $p > 0.3$), which was not apparent when using the full dataset.

The genetic similarity between pairs of individuals increased with time when basing the analyses on the subsets (Figure S12), corroborating the results seen with the full dataset (Figure 3, c), although not in a significant relationship in Central subsets. Again, differences between the results from the two subsampling approaches (‘geographic proximity’ vs. ‘random’) appeared minimal.

Temporal change of haplotype frequencies: exclusion of samples

Before constructing the haplotype networks, nine of the 94 generated mtDNA haplotype sequences were excluded from further analyses due to ambiguous or missing locality information. Furthermore, we excluded all samples that had a domestic cat haplotype ($n = 12$; haplotypes: H13, H16, H26, H32) and were classified as domestic cats based on the 10 HYB SNPs included in the SNP panel (available for 8 out of 12 excluded samples).

Current status of re-expanding wildcat populations

In a previous study, the species’ historical distribution was reviewed by means of a comprehensive research in literature, hunting statistics and regional archives (Reinert, 2017). When comparing this historical data (Figure 6, dark gray) to the available national monitoring data of contemporary wildcat occurrences (shades of green; Birlenbach et al., 2009; EEA, Art.17: European Environment Agency, 2020), large parts of the ranges’ core areas are congruent (Figure 6). The current monitoring under the habitats directive clearly illustrates the ongoing trend of species re-expansion, which appears to be rapidly recolonizing its former habitats (Balzer et al., 2018). Even so, some of the historical habitats are still abandoned or only sparsely populated to date, e.g. in the Black Forest and Swabian Alb region in Southwestern Germany (for geographical reference, compare Figure S2).

Supplementary Figures

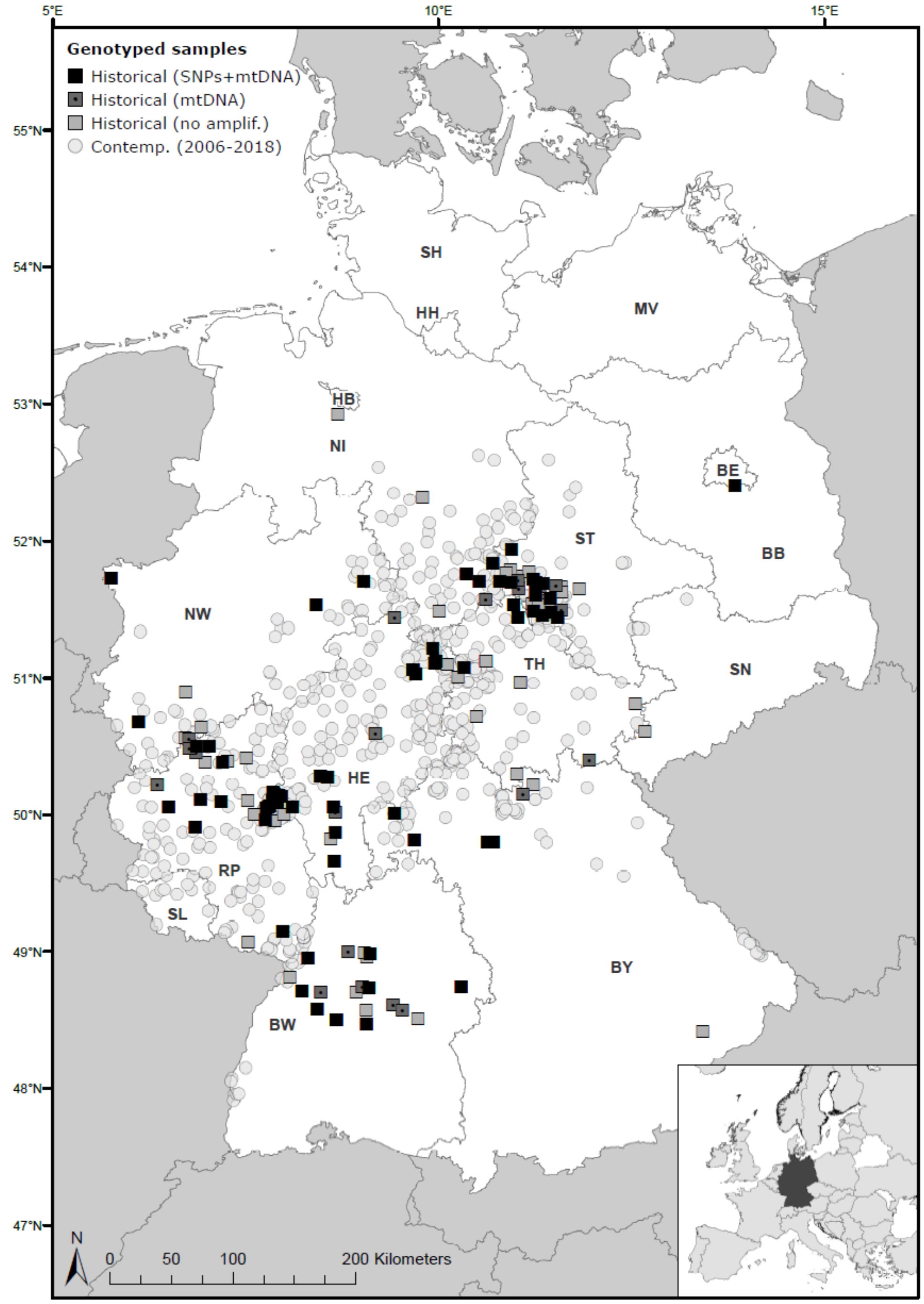

Figure S 1: Geographical distribution of genotyped wildcat samples used in this study. Light gray circles represent contemporary samples (*n* = 650; 2006-2018), while all squares represent historical samples from museum collections (*n* = 175; 1830-2001). Thereof, black squares represent samples with complete SNP and mtDNA data (*n* = 71), dark gray squares with dots represent samples with mtDNA data only (missing SNP genotypes, *n* = 15) and medium gray squares represent samples that have failed in genotyping. German federal states are labeled with acronyms: BW, Baden-Württemberg; BY, Bavaria; BE, Berlin; BB, Brandenburg; HB, Bremen; HH, Hamburg; HE, Hesse; MV, Mecklenburg-Vorpommern; NI, Lower Saxony; NW, North Rhine-Westphalia; RP, Rhineland-Palatinate; SL, Saarland; SN, Saxony; ST, Saxony-Anhalt; SH, Schleswig-Holstein; TH, Thuringia.

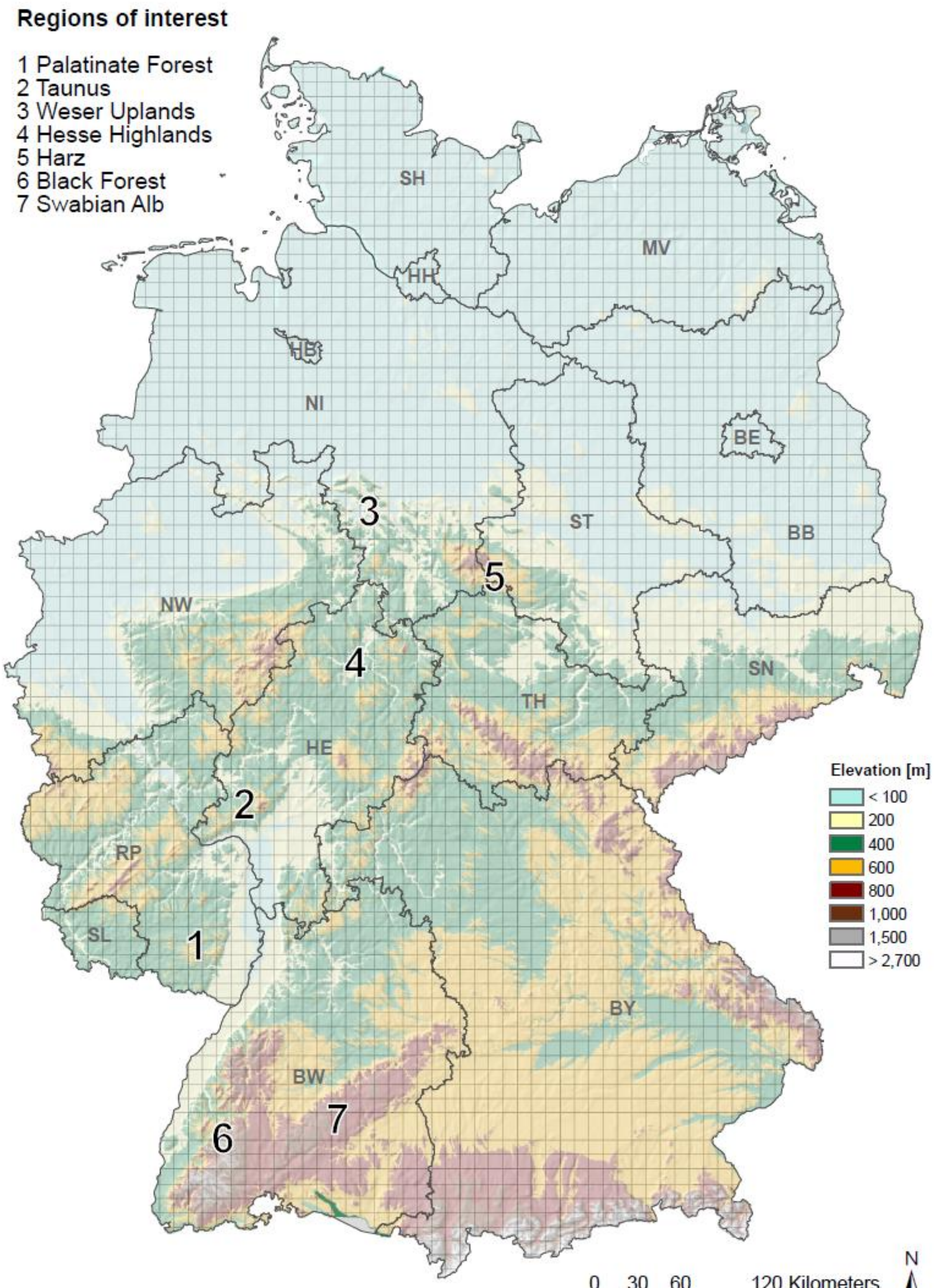

Figure S 2: Geographical regions of interest in this study. German federal states are labeled with acronyms: BW, Baden-Württemberg; BY, Bavaria; BE, Berlin; BB, Brandenburg; HB, Bremen; HH, Hamburg; HE, Hesse; MV, Mecklenburg-Vorpommern; NI, Lower Saxony; NW, North Rhine-Westphalia; RP, Rhineland-Palatinate; SL, Saarland; SN, Saxony; ST, Saxony-Anhalt; SH, Schleswig-Holstein; TH, Thuringia.

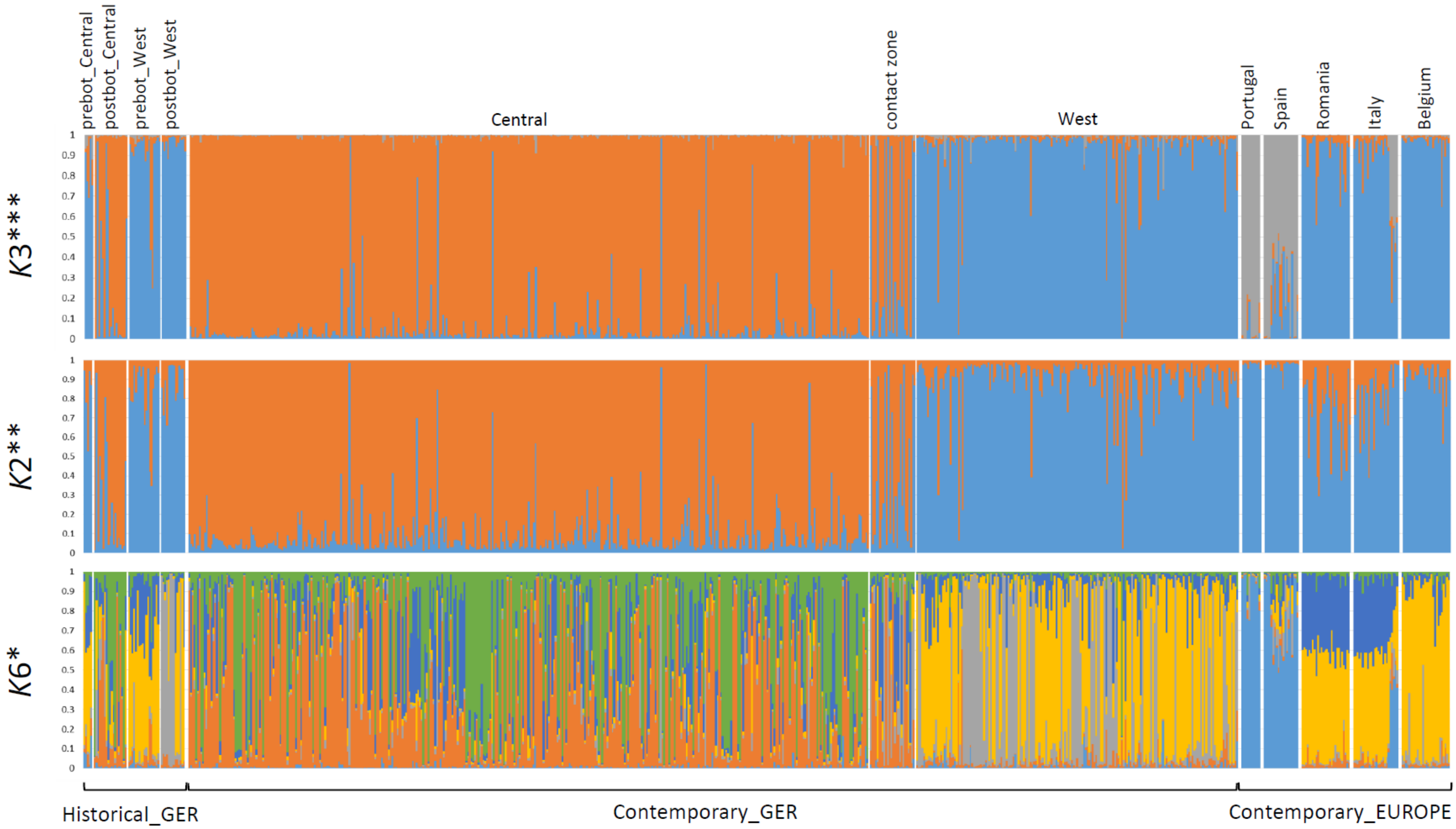

Figure S 3: Genetic population structure results for historical German populations and contemporary Europe-wide populations of wildcats. Genotypes of other European populations were taken from von Thaden et al., 2020. Each individual sample is represented by a vertical bar, in which colors indicate the likelihood of assignment ($q^{(i)}$) to the inferred genetic clusters. ***, most likely *K*; **, second most likely *K*; *, third most likely *K* (as calculated with the Evanno method, based on genotypes of 84 SNPs). prebot, pre-bottleneck; postbot, post-bottleneck; contact zone; hybrid zone between metapopulations (West, Central); GER, Germany.

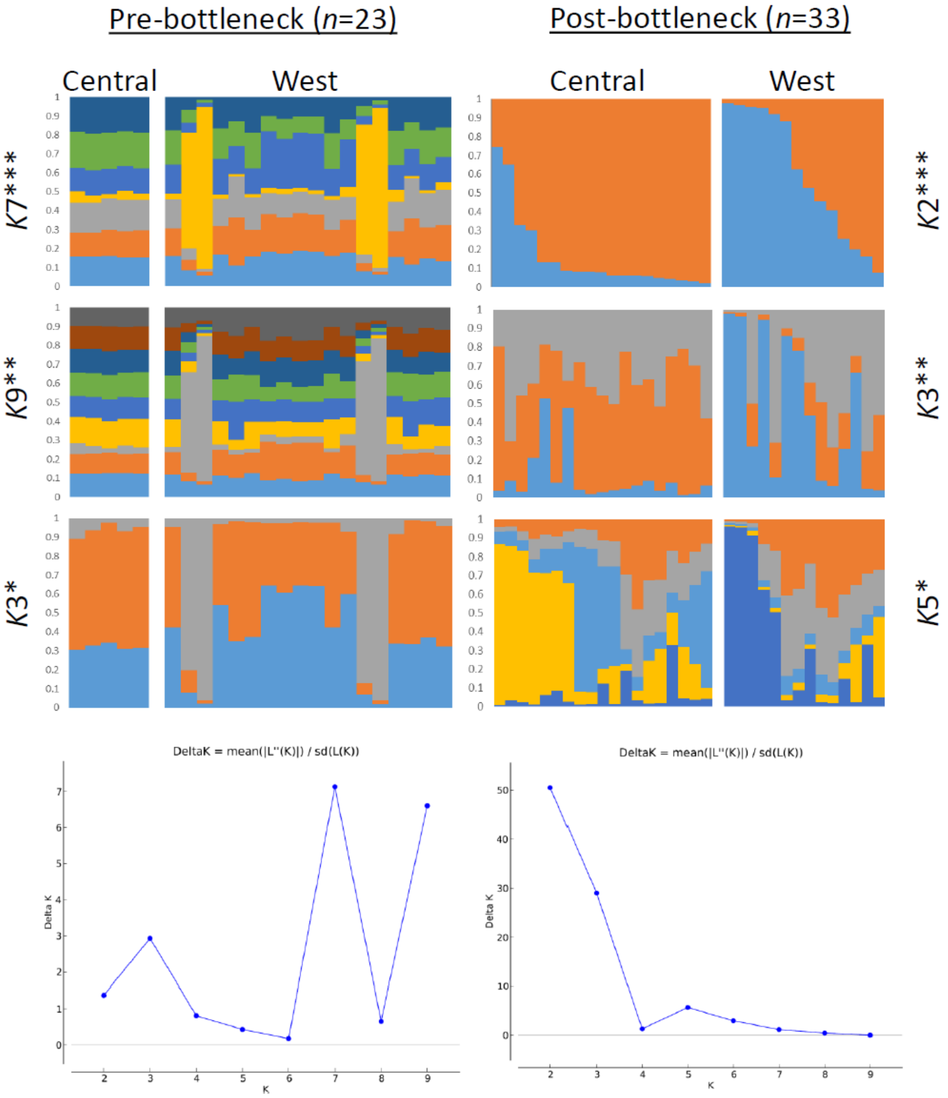

Figure S 4: Genetic population structure results for historical German wildcat populations. Each individual sample is represented by a vertical bar, in which colors indicate the likelihood of assignment ($q^{(i)}$) to the inferred genetic clusters. ***, most likely *K*; **, second most likely *K*; *, third most likely *K*. Bottom: Likelihood of *K*s calculated with the Evanno method based on genotypes of 84 SNPs.

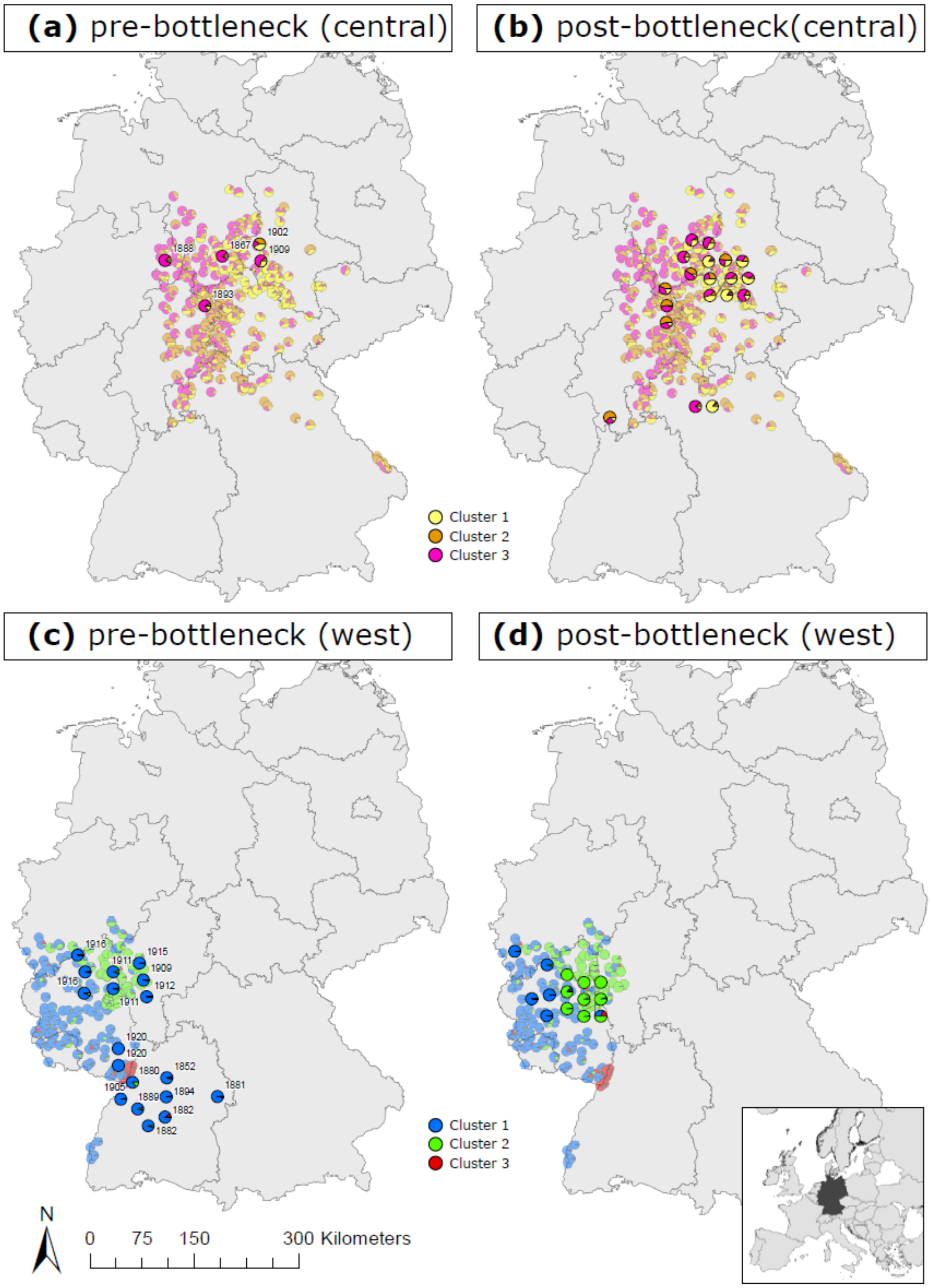

Figure S 5: Spatial genetic population structure for historical and contemporary German wildcat samples, separated by metapopulation. Results are shown separately for historical samples from pre- (a, c) and post-bottleneck (b, d) periods, with results for extant wildcats (Central, n = 395; West, n = 187) shown in the background of each map as a reference (transparent pie charts). Extant samples from the contact zone of the two metapopulations (n = 26) were excluded. Each individual is represented by a pie chart, in which colors indicate the likelihood of assignment ($q^{(i)}$) to the inferred genetic clusters. K = 3 was identified as the most likely K as calculated with the Evanno method based on the genotypes of 84 SNPs. Years in (a, c) correspond to the year of sample origin.

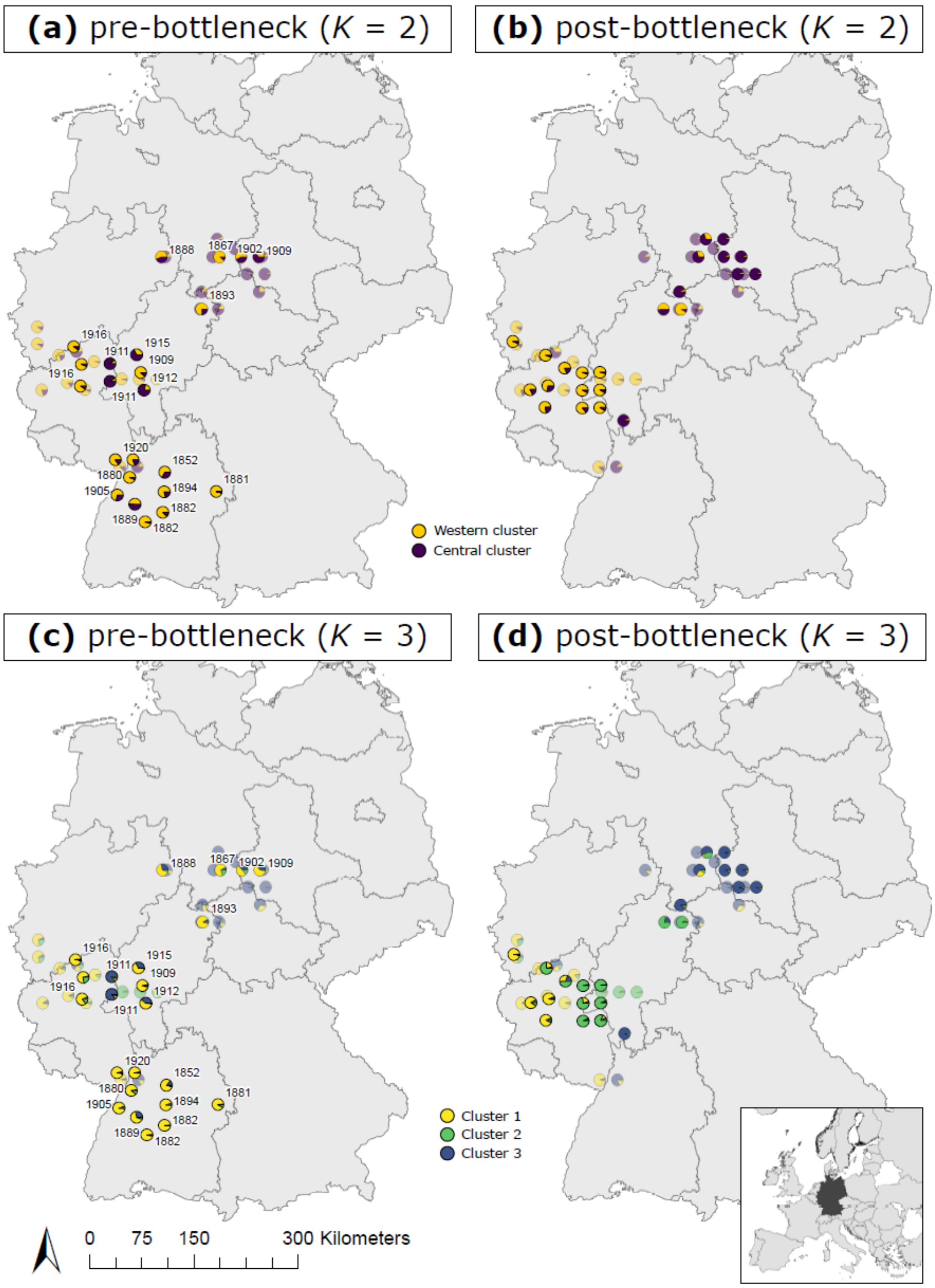

Figure S 6: Spatial genetic population structure for 'geographic proximity' subsets of historical and contemporary German wildcat samples. Sample sets were adjusted to an even sample size of n = 23 from each time period. Subsets from post-bottleneck and contemporary samples were selected based on geographic proximity. Results are shown separately for historical samples from pre- (a, c) and post-bottleneck (b, d) periods, with results for extant wildcats shown in the background of each map as a reference (transparent pie charts). Each individual is represented by a pie chart, in which colors indicate the likelihood of assignment ($q^{(i)}$) to the inferred genetic clusters. K = 3 was identified as the most likely K (c, d) as calculated with the Evanno method based on the genotypes of 84 SNPs, followed by the second most likely K = 2 (a, b). Years in (a, c) correspond to the year of sample origin.

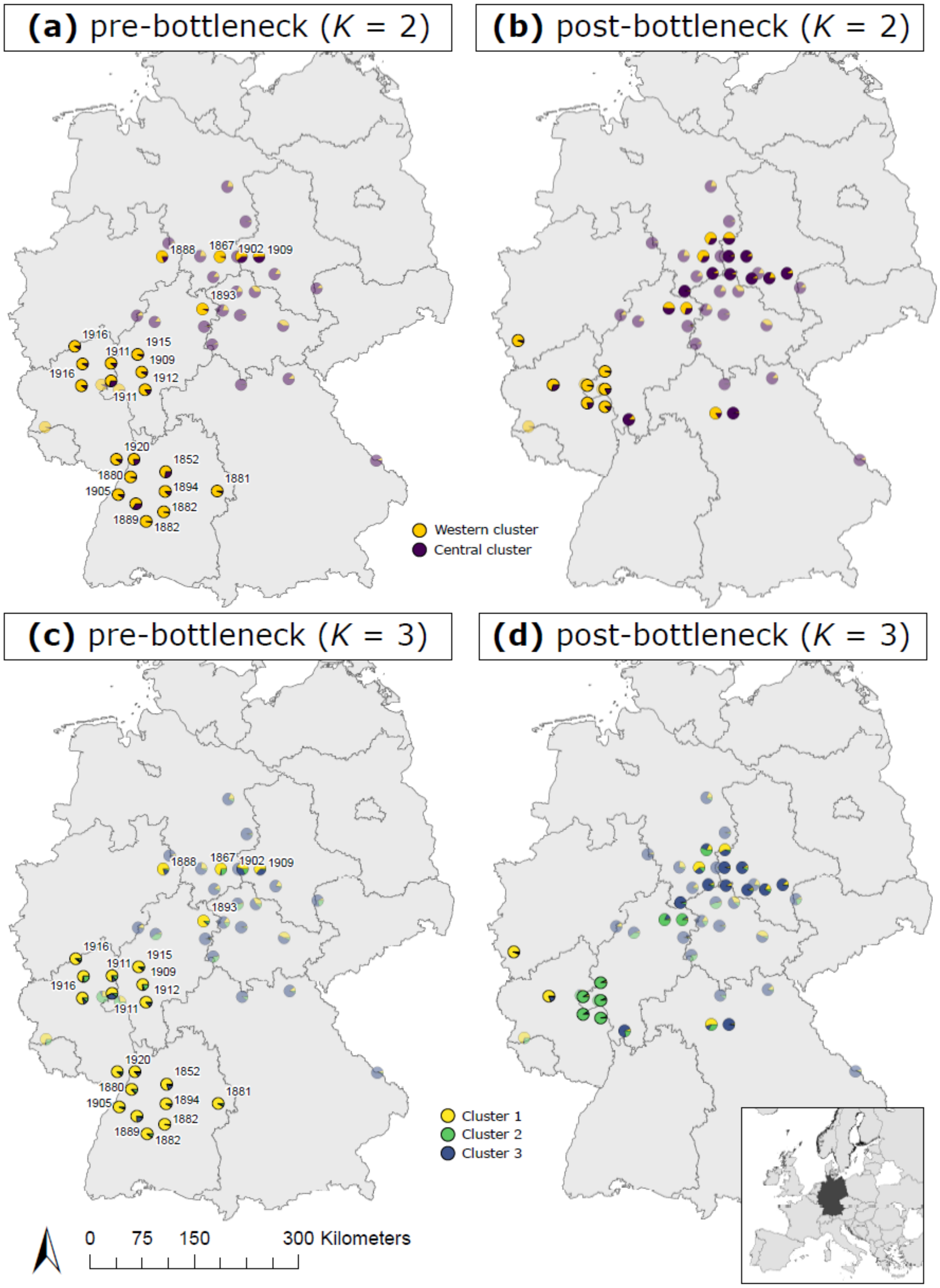

Figure S 7: Spatial genetic population structure for 'random' subsets of historical and contemporary German wildcat samples. Sample sets were adjusted to an even sample size of n = 23 from each time period. Subsets from post-bottleneck and contemporary samples were selected randomly. Results are shown separately for historical samples from pre- (a, c) and post-bottleneck (b, d) periods, with results for extant wildcats shown in the background of each map as a reference (transparent pie charts). Each individual is represented by a pie chart, in which colors indicate the likelihood of assignment ($q^{(i)}$) to the inferred genetic clusters. K = 2 was identified as the most likely K (a, b) as calculated with the Evanno method based on the genotypes of 84 SNPs, followed by the second most likely K = 3 (c, d). Years in (a, c) correspond to the year of sample origin.

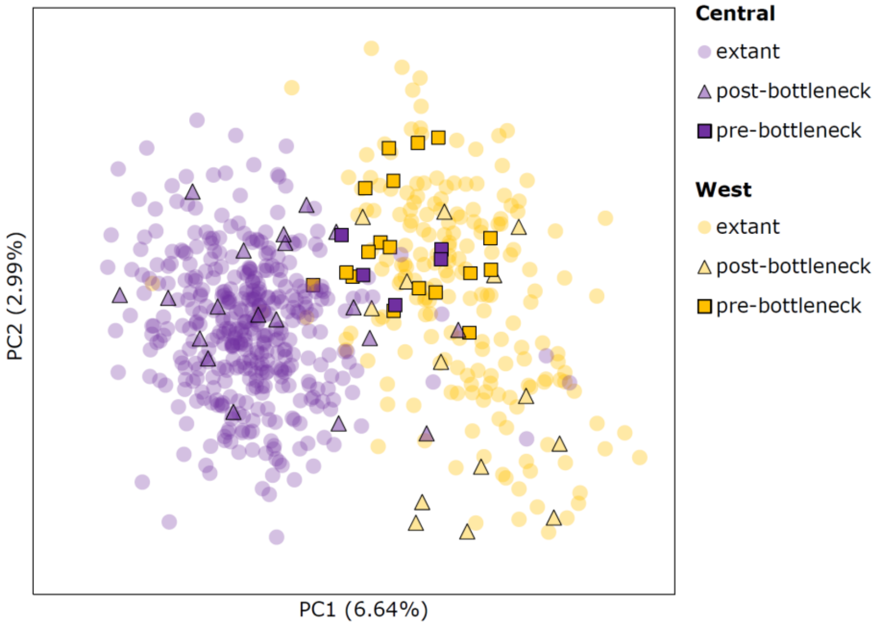

Figure S 8: Principal Coordinates Analysis (PCoA) of temporal and geographical groups. Samples were grouped into Central or West based on geographic sample origin. Temporal groups were assigned based on sampling date into extant (2006-2016), post-bottleneck (1950-2000) and pre-bottleneck (1852-1930). Each point, triangle and square represents an individual's genotype, color-coded to its sampling region (Central, purple; West, yellow) or temporal period (extant, circles; post-bottleneck, triangles; pre-bottleneck, squares).

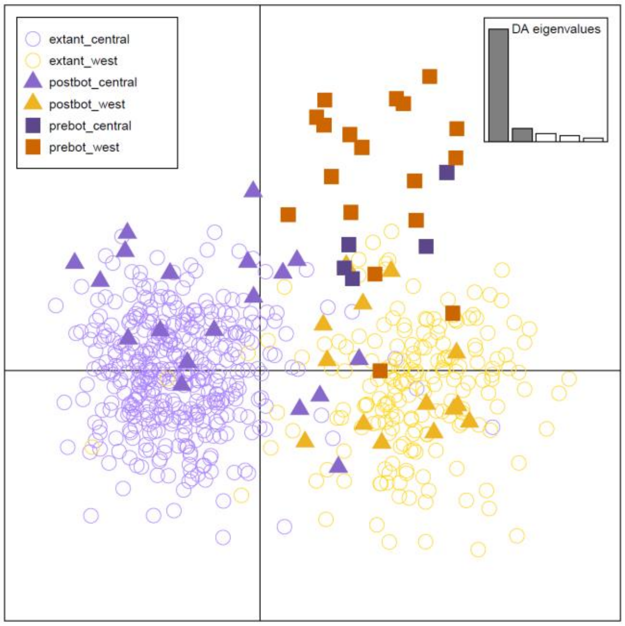

Figure S 9: Discriminant analysis of principal components (DAPC). Samples were grouped into Central or West based on geographic sample origin. Temporal groups were assigned based on sampling date into extant (2006-2016), post-bottleneck (1950-2000) and pre-bottleneck (1852-1930). Each point represents an individual's genotype, color-coded to its sampling region (Central, purple; West, yellow) or temporal period (extant, circles; post-bottleneck, triangles; pre-bottleneck, squares).

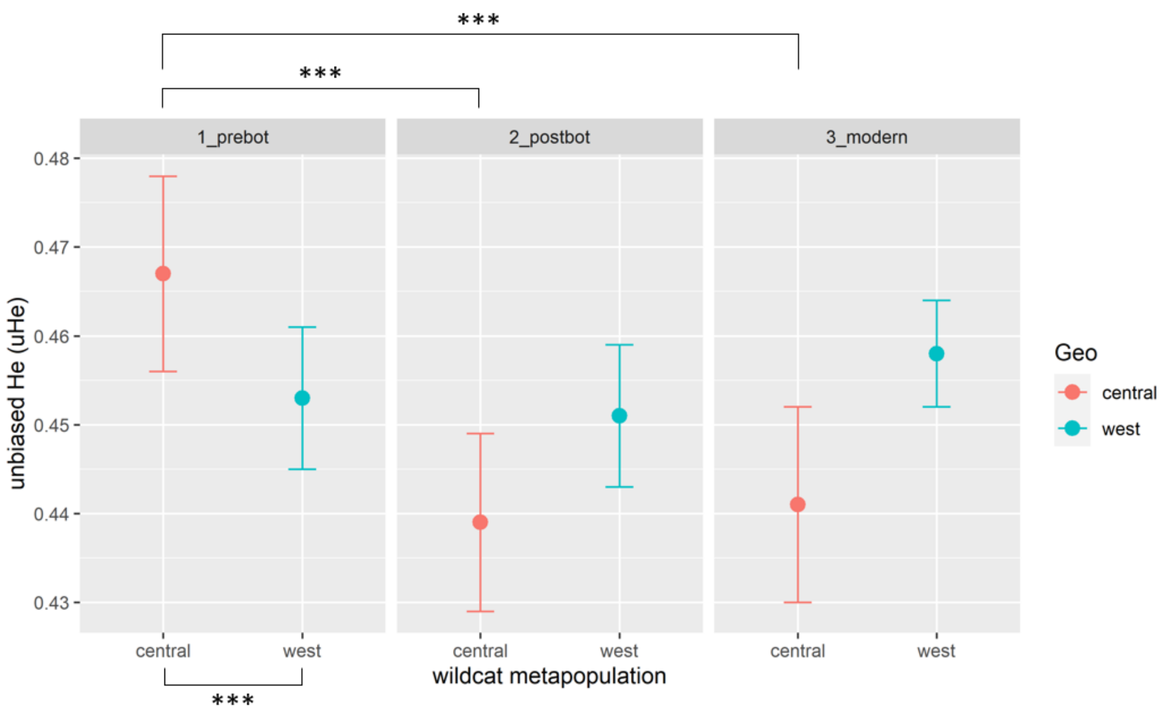

Figure S 10: Genetic diversity across periods. Points represent the average and standard error for measures of unbiased expected heterozygosity in each population. *** Highly significant difference, *p*-value < 0.001. prebot, pre-bottleneck; postbot, post-bottleneck; modern, extant. Based on full dataset (for sample numbers, compare Table S 4).

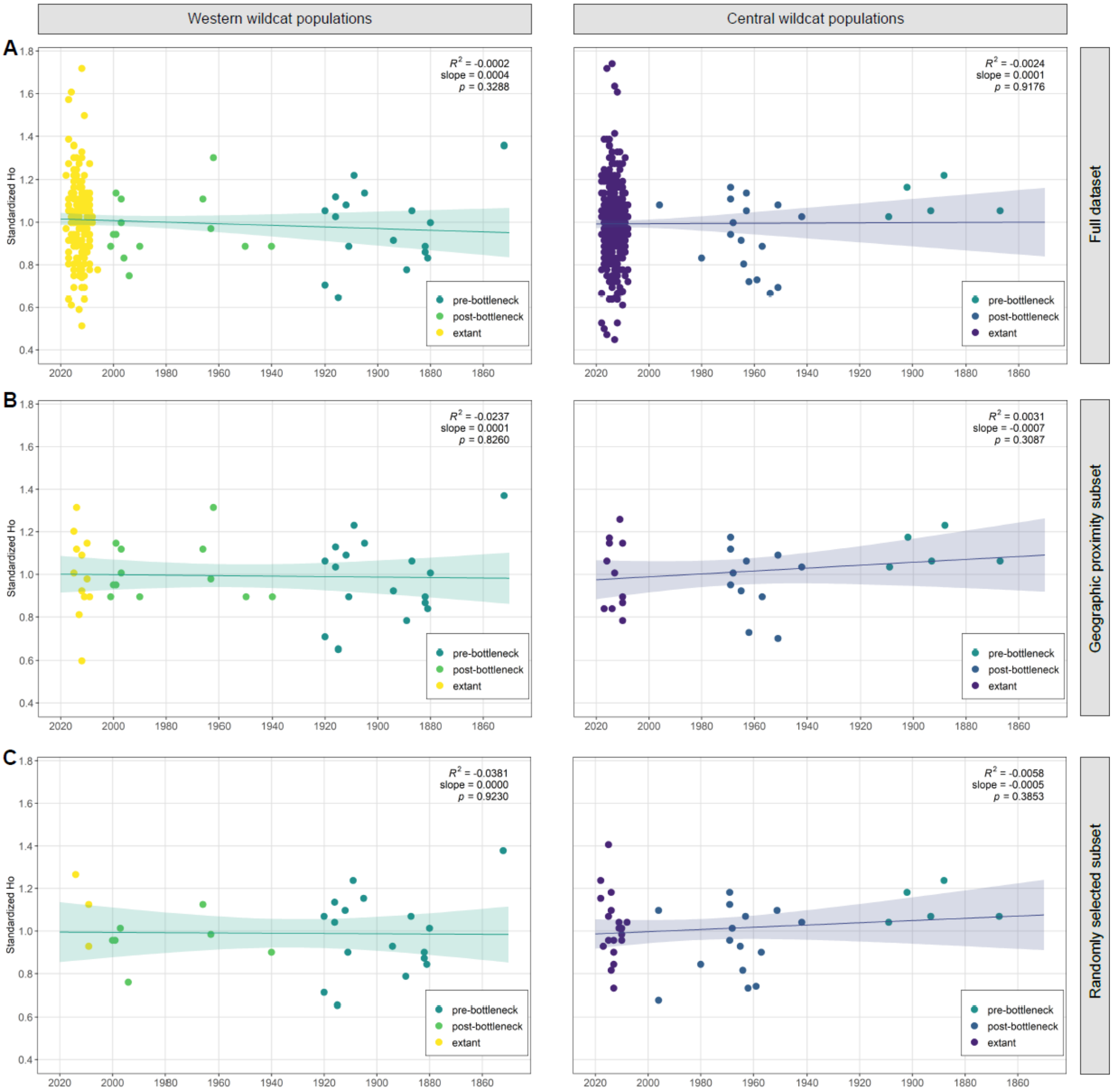

Figure S 11: Genetic diversity in German wildcat populations over time. (A) Individual standardized observed heterozygosity (H_O) for extant and historical wildcat populations over time, based on the full dataset (West: pre-bottleneck (pre-BN) n = 18, post-bottleneck (post-BN) n = 14, extant n = 187; Central: pre-BN n = 5, post-BN n = 19, extant n = 395). (B) Individual H_O for a subset of samples which was adjusted to an even sample size of n = 23 from each time period. Samples from post-BN and extant period were selected based on geographic proximity to pre-BN samples. (C) Individual H_O for a subset of samples which was adjusted to an even sample size of n = 23 from each time period. Samples from post-BN and extant period were selected randomly. For detailed sample numbers refer to Table S4.

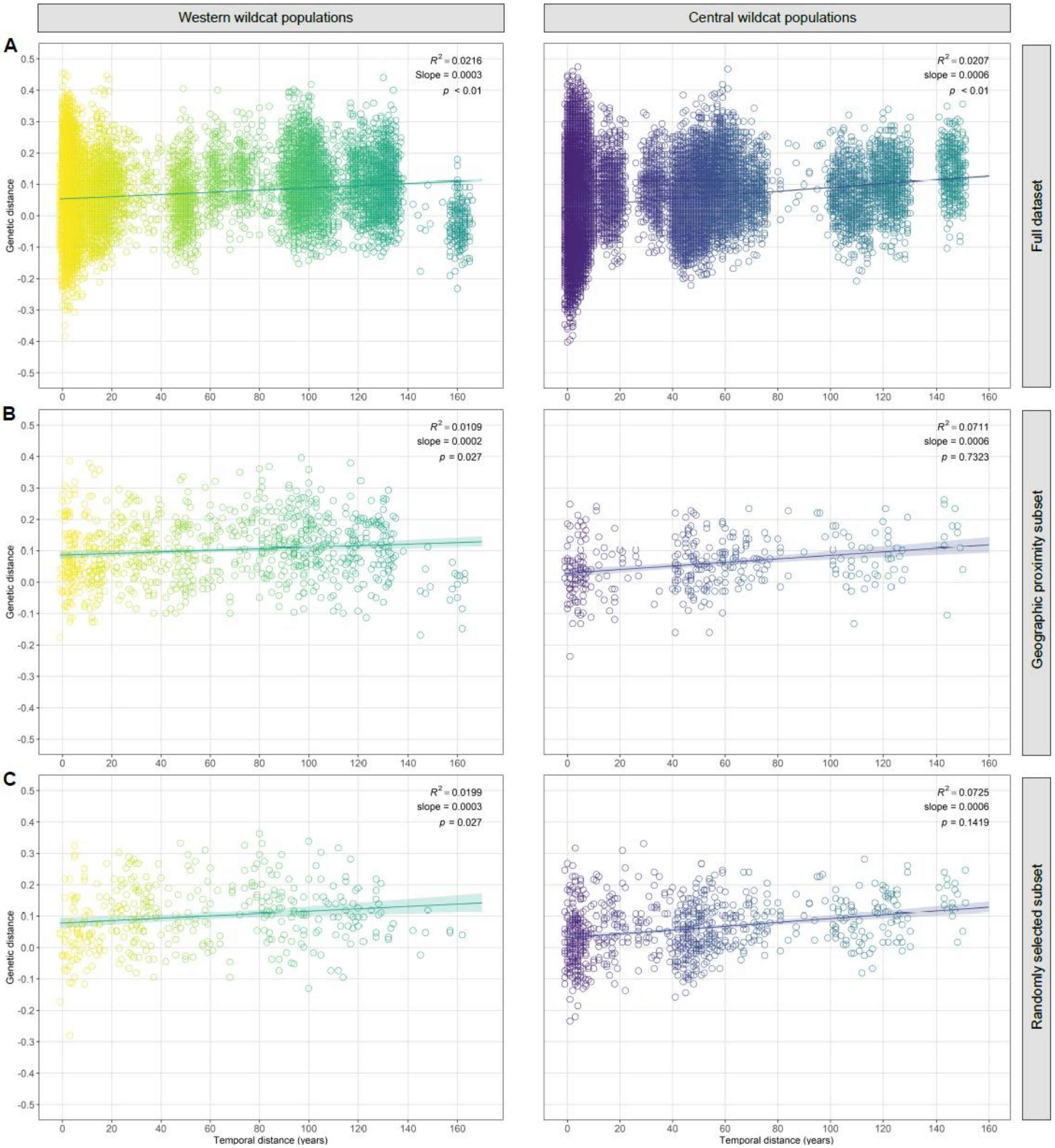

Figure S 12: Genetic distance in German wildcat populations over time. (A) Genetic versus temporal distance for pairs of individual samples based on the full dataset (West: pre-bottleneck (pre-BN) n = 18, post-bottleneck (post-BN) n = 14, extant n = 187; Central: pre-BN n = 5, post-BN n = 19, extant n = 395). (B) Genetic versus temporal distance based on a subset of samples that was adjusted to an even sample size of n = 23 from each time period. Samples from post-BN and extant period were selected based on geographic proximity to pre-BN samples. (C) Genetic distance versus temporal distance based on a subset of samples that was adjusted to an even sample size of n = 23 from each time period. Samples from post-BN and extant period were selected randomly. For detailed sample numbers refer to Table S4.

Supplementary Tables

Table S 1: Samples of historical and contemporary European wildcats used in this study.

Sample set	Type of samples	*n*
Historical	*German wildcat museum samples*	175
	Thereof with known locality	148
	Complete mtDNA data	85[a]
	Excluded due to domestic cat haplotype	12
	Used for mtDNA analyses	73
	Pre-bottleneck	33
	West	24
	Central	9
	Post-bottleneck	40
	West	17
	Central	23
	Sufficient SNP data	71[b]
	Thereof domestic cats	9
	Thereof putative hybrids	6
	Thereof wildcats (used for analyses)	56
	Pre-bottleneck	23
	West	18
	Central	5
	Post-bottleneck	33
	West	14
	Central	19
Contemporary	*German wildcat monitoring samples*	650
	Thereof domestic cats	2
	Thereof putative hybrids	20
	Thereof q^{wc}<0.85	20
	Thereof wildcats	608

[a] 94 samples with complete mtDNA data, but 9 samples with uncertain locality were excluded.
[b] 76 samples with sufficient SNP data, but 5 samples with uncertain locality were excluded.

Table S 2: F_{ST} values for the different *K* runs and obtained clusters in STRUCTURE. Individuals not assigned to a cluster with $q^{(i)} \leq 0.8$ were excluded. All obtained values are highly significant ($p < 0.001$).

		West_*K*3			Central_*K*3			All_*K*2		All_*K*5					
		Q1_Rhine_South	Q2_Rhine_West	Q3_Rhine_East	Q1_Hesse_Highlands	Q2_Weser_Uplands	Q3_Harz	Q1_Central	Q2_West	Q1_Rhine_East	Q2_Rhine_West	Q3_Weser_Uplands	Q4_Hesse_Highlands	Q5_Harz	
West_*K*3	Q1_Rhine_South	-													*n*=13
	Q2_Rhine_West	0.12	-												*n*=116
	Q3_Rhine_East	0.20	0.06	-											*n*=61
Central_*K*3	Q1_Hesse_Highlands				-										*n*=36
	Q2_Weser_Uplands				0.08	-									*n*=33
	Q3_Harz				0.10	0.09	-								*n*=40
All_*K*2	Q1_Central							-							*n*=379
	Q2_West							0.07	-						*n*=215
All_*K*5	Q1_Rhine_East									-					*n*=62
	Q2_Rhine_West									0.07	-				*n*=107
	Q3_Weser_Uplands									0.14	0.09	-			*n*=34
	Q4_Hesse_Highlands									0.13	0.08	0.07	-		*n*=92
	Q5_Harz									0.16	0.10	0.08	0.06	-	*n*=67

Table S 3: Percentages of molecular variance as determined with AMOVA. Based on the full dataset (compare to Table S1).

	Pre- vs. Post-bottleneck		Post-bottleneck vs. Extant		West vs. Central metapopulation		
	West	Central	West	Central	Pre-bottleneck	Post-bottleneck	Extant
among populations	4%	4%	1%	1%	2%	6%	6%
among individuals	8%	7%	5%	3%	7%	8%	3%
between individuals	88%	89%	94%	95%	91%	86%	91%

Table S 4: Subsets of wildcat samples used to test for potential sampling bias. Sample sets were adjusted to an even sample size of n = 23 from each time period based on the number of available pre-bottleneck (Pre-BN) samples in this study. For the geographic proximity subset, samples from the post-bottleneck (Post-BN) and contemporary (extant) period were selected bases on geographic proximity to the pre-BN samples. For the randomly selected subset, samples from the Post-BN and extant period were subsampled randomly, using the *sample()* function in R.

		Pre-BN	Post-BN	Extant
Full dataset	West	18	14	187
	Central	5	19	395
	Total *n*	23	33	582[a]
Geographic proximity subset	West	18	12	12
	Central	5	11	11
	Total *n*	23	23	23
Randomly selected subset	West	18	7	3
	Central	5	16	20
	Total *n*	23	23	23

[a] 608 of the 650 contemporary samples were identified as pure wildcats (compare Table S1) and 26 samples from the extant contact zone of the two metapopulations were excluded for analyses in which Central and West were assessed separately.

Table S 5: Pairwise genetic differentiation between temporal and geographical groups. Sample sets were adjusted to an even sample size of n = 23 from each time period and subsets were selected based on geographic proximity. Pairwise F_{ST} values below diagonal, p-values above diagonal. F_{ST} values were assumed as significantly different from zero for $p < 0.05$; based on 5,000 permutations. Pre-BN; pre-bottleneck; Post-BN; post-bottleneck.

		West			Central		
		Pre-BN	Post-BN	Extant	Pre-BN	Post-BN	Extant
West	Pre-BN (*n*=18)	-	0.00	0.00	0.07	0.00	0.00
	Post-BN (*n*=12)	0.03	-	0.39	0.01	0.00	0.00
	Extant (*n*=12)	0.03	0.00	-	0.11	0.00	0.00
Central	Pre-BN (*n*=5)	0.02	0.04	0.02	-	0.00	0.00
	Post-BN (*n*=11)	0.07	0.06	0.06	0.04	-	0.01
	Extant (*n*=11)	0.05	0.06	0.05	0.05	0.02	-

Table S 6: Pairwise genetic differentiation between temporal and geographical groups. Sample sets were adjusted to an even sample size of n = 23 from each time period and subsets were selected randomly. Pairwise F_{ST} values below diagonal, p-values above diagonal. F_{ST} values were assumed as significantly different from zero for $p < 0.05$; based on 5,000 permutations. Pre-BN; pre-bottleneck; Post-BN; post-bottleneck.

		West			Central		
		Pre-BN	Post-BN	Extant	Pre-BN	Post-BN	Extant
West	Pre-BN (*n*=18)	-	0.00	0.10	0.07	0.00	0.00
	Post-BN (*n*=7)	0.05	-	0.68	0.03	0.00	0.00
	Extant (*n*=3)	0.03	-0.01	-	0.12	0.02	0.00
Central	Pre-BN (*n*=5)	0.02	0.06	0.03	-	0.01	0.00
	Post-BN (*n*=16)	0.06	0.07	0.06	0.04	-	0.05
	Extant (*n*=20)	0.06	0.07	0.08	0.06	0.01	-

Table S 7: Historical and contemporary SNP diversity and differentiation in German wildcat populations. Sample sets were adjusted to an even sample size of n = 23 from each time period and subsets were selected based on geographic proximity. n, number of individuals; H_O, observed heterozygosity; uH_E, unbiased expected heterozygosity; F_{IS}, population inbreeding coefficient; F_{ST}, genetic differentiation coefficient. Pre-BN; pre-bottleneck; Post-BN; post-bottleneck.

Epoch	Meta-population	n	Dates range	H_O	uH_E	F_{IS}	p-value	F_{ST} global	p-value
Pre-BN		23	1852-1920	0.43 (± 0.01)	0.46 (± 0.01)	0.07	0.07	0.02	0.15
	West	18	1852-1920	0.42 (± 0.01)	0.45 (± 0.01)	0.08	0.08		
	Central	5	1867-1909	0.47 (± 0.02)	0.47 (± 0.01)	-0.02	0.54		
Post-BN		23	1940-2001	0.42 (± 0.01)	0.46 (± 0.01)	0.06	0.14	0.06	0.01
	West	12	1940-2001	0.43 (± 0.01)	0.46 (± 0.01)	0.06	0.19		
	Central	11	1942-1969	0.41 (± 0.02)	0.43 (± 0.01)	0.05	0.28		
Extant		23	2009-2017	0.43 (± 0.01)	0.47 (± 0.01)	0.07	0.05	0.05	0.01
	West	12	2009-2015	0.43 (± 0.01)	0.47 (± 0.01)	0.09	0.10		
	Central	11	2010-2017	0.43 (± 0.02)	0.44 (± 0.01)	0.04	0.30		

Table S 8: Historical and contemporary SNP diversity and differentiation in German wildcat populations. Sample sets were adjusted to an even sample size of n = 23 from each time period and subsets were selected randomly. n, number of individuals; H_O, observed heterozygosity; uH_E, unbiased expected heterozygosity; F_{IS}, population inbreeding coefficient; F_{ST}, genetic differentiation coefficient. Pre-BN; pre-bottleneck; Post-BN; post-bottleneck.

Epoch	Meta-population	n	Dates range	H_O	uH_E	F_{IS}	p-value	F_{ST} global	p-value
Pre-BN		23	1852-1920	0.43 (± 0.01)	0.46 (± 0.01)	0.07	0.07	0.02	0.15
	West	18	1852-1920	0.42 (± 0.01)	0.45 (± 0.01)	0.08	0.08		
	Central	5	1867-1909	0.47 (± 0.02)	0.47 (± 0.01)	-0.02	0.54		
Post-BN		23	1940-2000	0.41 (± 0.01)	0.46 (± 0.01)	0.09	0.04	0.07	0.00
	West	7	1940-2000	0.40 (± 0.02)	0.44 (± 0.01)	0.09	0.09		
	Central	16	1942-1996	0.41 (± 0.02)	0.44 (± 0.01)	0.08	0.07		
Extant		23	2008-2018	0.44 (± 0.01)	0.45 (± 0.01)	0.01	0.45	0.08	0.02
	West	3	2009-2014	0.47 (± 0.03)	0.45 (± 0.02)	-0.05	0.66		
	Central	20	2008-2018	0.43 (± 0.02)	0.44 (± 0.01)	0.02	0.38		

Table S 9: Percentages of molecular variance as determined with AMOVA. Sample sets were adjusted to an even sample size of *n* = 23 from each time period and subsets were selected based on geographic proximity (compare to Table S4).

	Pre- vs. Post-bottleneck		Post-bottleneck vs. Extant		West vs. Central metapopulation		
	West	Central	West	Central	Pre-bottleneck	Post-bottleneck	Extant
among populations	3%	4%	0%	2%	2%	6%	5%
among individuals	8%	3%	8%	5%	7%	5%	7%
between individuals	89%	93%	92%	93%	91%	89%	88%

Table S 10: Percentages of molecular variance as determined with AMOVA. Sample sets were adjusted to an even sample size of *n* = 23 from each time period and subsets were selected randomly (compare to Table S4).

	Pre- vs. Post-bottleneck		Post-bottleneck vs. Extant		West vs. Central metapopulation		
	West	Central	West	Central	Pre-bottleneck	Post-bottleneck	Extant
among populations	5%	4%	0%	1%	2%	7%	8%
among individuals	9%	6%	5%	5%	7%	8%	1%
between individuals	86%	91%	95%	94%	91%	85%	91%

References

Balzer, S., Mölich, T., Streif, S., Tiesmeyer, A., Thein, J., Nowak, C. (2018). Status der Wildkatze in Deutschland. *Natur und Landschaft, 93*(4), 146–152. https://doi.org/10.17433/4.2018.50153561.146-152

Birlenbach, K., Klar, N. (2009). Aktionsplan zum Schutz der Europäischen Wildkatze in Deutschland. *Naturschutz und Landschaftsplanung, 41*(11), 325–332.

European Environment Agency (2020). State of nature in the EU: results from reporting under the nature directives 2013-2018. https://doi.org/10.2800/088178

Reinert, K. (2017). Eine GIS-basierte Rekonstruktion der historischen Verbreitung von Wolf (*Canis lupus*) und Wildkatze (*Felis silvestris silvestris*) in Deutschland: Implikationen für das Wildtiermanagement. Frankfurt am Main. Johann Wolfgang Goethe-Universität. 123 p.

von Thaden, A., Nowak, C., Tiesmeyer, A., Reiners, T. E., Alves, P. C., Lyons, L. A., Mattucci, F., Randi, E., Cragnolini, M., Galián, J., Hegyeli, Z., Kitchener, A. C., Lambinet, C., Lucas, J. M., Mölich, T., Ramos, L., Schockert, V., Cocchiararo, B. (2020). Applying genomic data in wildlife monitoring: Development guidelines for genotyping degraded samples with reduced single nucleotide polymorphism (SNP) panels. *Molecular Ecology Resources, 20*(3), 662–680. https://doi.org/10.1111/1755-0998.13136

Wahlund, S. (1928). Composition of populations and correlation appearances viewed in relation to the studies of inheritance. *Hereditas*, 11, 65–106.
